# Optical Hydrogen Nanothermometry of Plasmonic Nanoparticles under Illumination

**DOI:** 10.1021/acsnano.2c00035

**Published:** 2022-03-28

**Authors:** Christopher Tiburski, Ferry Anggoro Ardy Nugroho, Christoph Langhammer

**Affiliations:** † Department of Physics, Chalmers University of Technology, 412 96 Göteborg, Sweden; ‡ Department of Physics and Astronomy, Vrije Universiteit Amsterdam, De Boelelaan 1081, 1081 HV Amsterdam, The Netherlands

**Keywords:** nanoparticles, plasmonics, nanothermometry, sensing, palladium hydride, temperature, photothermal

## Abstract

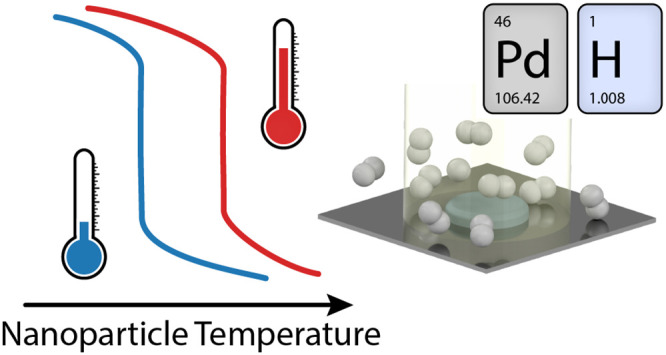

The temperature of nanoparticles is a critical parameter in applications that range from biology, to sensors, to photocatalysis. Yet, accurately determining the absolute temperature of nanoparticles is intrinsically difficult because traditional temperature probes likely deliver inaccurate results due to their large thermal mass compared to the nanoparticles. Here we present a hydrogen nanothermometry method that enables a noninvasive and direct measurement of absolute Pd nanoparticle temperature *via* the temperature dependence of the first-order phase transformation during Pd hydride formation. We apply it to accurately measure light-absorption-induced Pd nanoparticle heating at different irradiated powers with 1 °C resolution and to unravel the impact of nanoparticle density in an array on the obtained temperature. In a wider perspective, this work reports a noninvasive method for accurate temperature measurements at the nanoscale, which we predict will find application in, for example, nano-optics, nanolithography, and plasmon-mediated catalysis to distinguish thermal from electronic effects.

Metal nanoparticles have found application in a wide range of fields owing to their support of localized surface plasmon resonances (LSPRs).^1,2^ All these applications have in common that they rely on the irradiation of light to unlock plasmonic functions utilized, for example, for optical sensing,^3,4^ treatment of disease,^5,6^ photovoltaic devices,^7,8^ optical metamaterials,^9−11^ dynamic coloring,^12,13^ and active plasmonics^14^ and to enhance catalytic reactions.^15−17^ At the same time, it is well known that LSPR excitation leads to significant light absorption, which *via* coupling to lattice phonons increases nanoparticle temperature,^18−20^ and that such a temperature increase may have both wanted^21−23^ and unwanted^24^ consequences for specific applications. One such area where the potential impact of plasmon-induced optical heating of nanoparticles is highly debated^25−30^ and potentially very significant^31^ is plasmon-mediated catalysis. In this field, several different reaction enhancement mechanisms have been proposed,^31−34^ and it remains a great challenge to distinguish between photothermal- and hot carrier-induced reaction enhancement.^35^ One reason is that commonly conducted experiments to disentangle these mechanisms rely on probing the catalytic activity as a function of photon flux. Such experiments have been demonstrated to yield ambiguous results since several orders of magnitude change of the photon flux would be needed for rigorous analysis,^35^ which experimentally is nearly impossible to achieve. Similarly, actual measurements of light-induced temperature changes in plasmonic systems is very challenging, especially in more complex systems and if the temperature probes used are not placed very carefully.^25^ This is the consequence of a combination of factors that include (i) the high thermal conductivity of metal nanoparticles that leads to rapid thermal equilibration with their support,^36^ (ii) their small thermal mass that renders macroscopic temperature probes highly invasive, and (iii) thermal gradients that readily occur, in particular in three-dimensional samples.^37^ As a consequence, *direct* and noninvasive measurements of plasmonic nanoparticle temperature *in situ* are highly desirable to resolve this long-standing challenge in plasmonic technologies in general^38−43^ and in plasmon-mediated catalysis in particular.^25−30^ Focusing on catalysis, several attempts in this direction have been reported. They include anti-Stokes radiation and fluorescence spectroscopy that either require rather complicated equipment or the use of fluorescent labels,^44−47^ or the application of a specific temperature-sensitive thin film coating to the sample,^48^ which renders it a destructive measurement method. These and other thermometry methods for the nano- and microscale can achieve in some cases high temporal resolution of down to 10^–9^ s, spatial resolutions of 10^–2^ μm, or temperature resolution of 10^–5^ K.^49^ However, although all of these are impressive achievements, a reasonably simple, noninvasive, and nondestructive means to *directly* measure the temperature of metal nanoparticles with good resolution in general, and of plasmonic nanoparticles under different levels of illumination in particular, still does not exist.

In response, we here discuss the concept of optical hydrogen nanothermometry, which enables direct and noninvasive temperature measurements of Pd nanoparticles under illumination. We utilize the method to unravel LSPR-induced nanoparticle heating effects at an absolute scale for different irradiances of incident visible light and nanoparticle arrays with different surface coverage, in combination with numerical simulations and theoretical modeling.

## Results and Discussion

**Figure 1:**
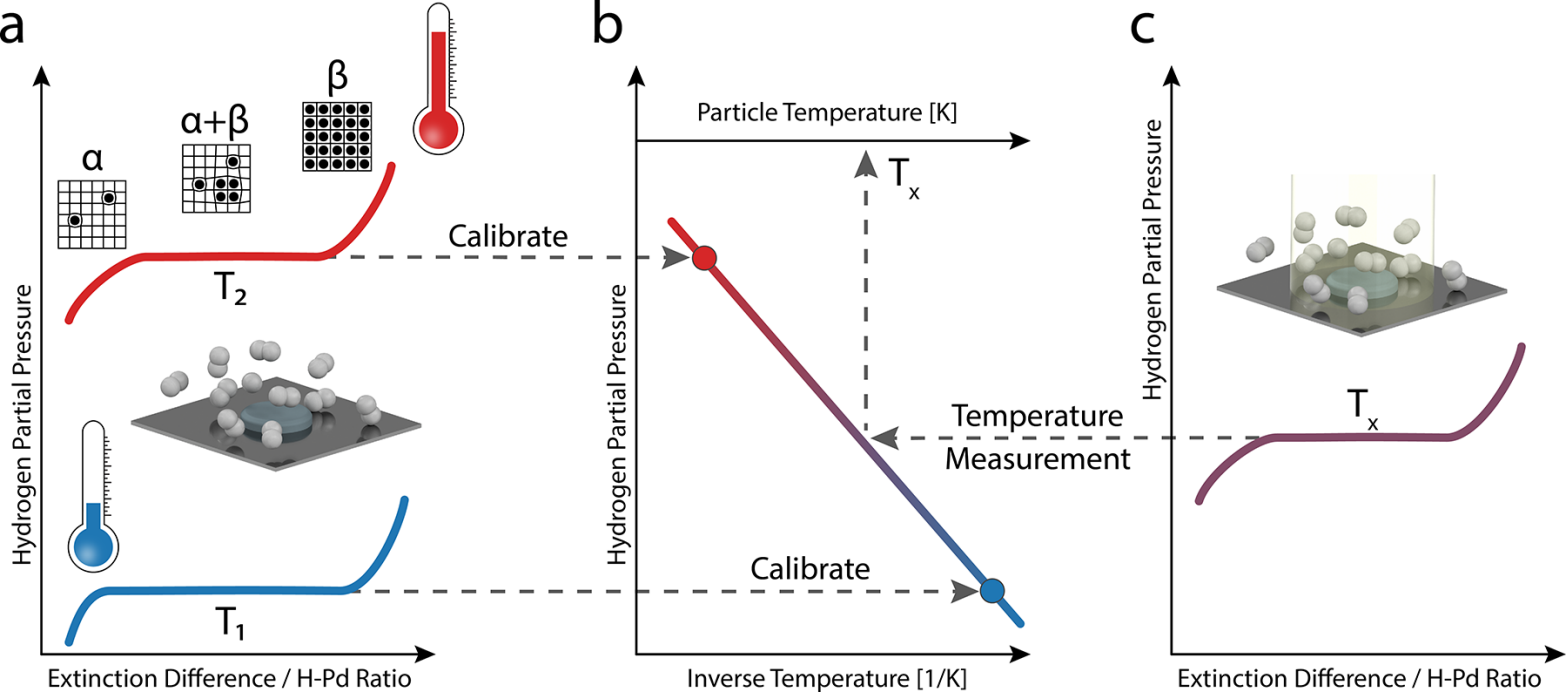
Hydrogen nanothermometry concept of using a Van’t Hoff plot to deduce the temperature of Pd nanoparticles. (a) Pressure–composition isotherms are a common way of characterizing metal–hydrogen interactions at different temperatures and enable, among others, the construction of a phase diagram. Specifically, for the Pd–H case at hand, a pressure–composition isotherm reveals the relation between external hydrogen (partial) pressure and the amount of hydrogen atoms occupying the interstitial lattice sites in the Pd host, *i*.*e*., the H/Pd ratio. At low hydrogen pressure the hydrogen atoms form a solid solution in the Pd lattice (α-phase). At a critical hydrogen pressure, due to now sizable H–H interaction, the hydride (β-phase) nucleates and both phases coexist, resulting in a “plateau” in the isotherm that signifies this first-order phase transformation. As the key point for our present work, this phase transition will shift to a different pressure at different temperature (*T*_1_, *T*_2_, where *T*_2_ > *T*_1_). (b) Van’t Hoff plot that depicts the phase transition pressure of the Pd–H system plotted on a semilogarithmic scale *versus* the inverse temperature exhibiting a linear relation. (c) Determination of an unknown temperature (*T*_*x*_) of a Pd nanoparticle system by correlating the obtained phase transition pressure with the premeasured Van’t Hoff plot.

When Pd, in general, and its nanoparticles, in particular, are exposed to hydrogen, they transform into a hydride as a consequence of dissociative chemisorption of hydrogen molecules on Pd surfaces and their subsequent diffusion into interstitial Pd lattice sites.^50,51^ At low hydrogen pressures, the absorbed hydrogen atoms form a solid solution in the Pd lattice (α-phase) where they are sparsely distributed and the H–H interactions are weak.^52^ At a critical pressure where hydrogen-induced electronic and strain-field interactions become sizable, the hydride (β-phase) nucleates.^52^ At this point both phases (α + β) coexist and the system undergoes a first-order phase transformation, which gives rise to a distinct “plateau” in a pressure–composition isotherm (Figure 1a). This phase transformation significantly changes the electronic structure and volume of the system,^53−55^ which enables its tracking using optical methods in both thin films^56,57^ and nanoparticles,^58^ where LSPR excitations are important in the latter case. To this end, it has also been demonstrated experimentally^58,59^ and theoretically from first-principles^60^ that various descriptors of the LSPR peak, such as shifts in the spectral position or, as we will use here, the extinction difference at two rationally selected wavelengths of self-referenced spectra,^61^ are proportional to the atomic ratio of hydrogen and Pd and therefore can be used to construct optical isotherms (Figure 1a).

The occurrence of the α- to β-phase transformation depends on the temperature of the system and is shifted to higher pressures when the temperature increases (Figure 1a). This temperature dependence is widely used to construct Van’t Hoff plots (Figure 1b) to extract the hydride formation enthalpy and entropy change according to the Van’t Hoff equation
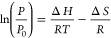
1where *P* is the is phase transition pressure, *P*_0_ is atmospheric pressure,  and  are the change in enthalpy and entropy, respectively, *R* is the universal gas constant, and *T* is temperature.

Here, we propose a different consequence of this intrinsic temperature dependence of the two-phase coexistence transition, namely, that a “reversed” Van’t Hoff analysis can be used to accurately measure the temperature of the hydrogenated system, provided it has been appropriately calibrated. Specifically, by first generating a Van’t Hoff plot at well-defined temperatures, it can subsequently be used to derive the temperature of the system *via* the phase transition pressure measured at that particular (unknown) temperature. Projecting this concept onto the case of interest here, that is, light-induced heating of plasmonic nanoparticles, it means that it can be used to accurately determine the *absolute* temperature of Pd nanoparticles under illumination (Figure 1c). As the key aspect of this approach, we highlight that the temperature obtained this way corresponds to the *true* nanoparticle temperature since it is only the Pd particles *themselves* that undergo the temperature-dependent hydride formation process. Hence, our concept enables noninvasive, nondestructive direct measurements of nanoparticle temperature changes induced by light absorption, as we will demonstrate below.

**Figure 2:**
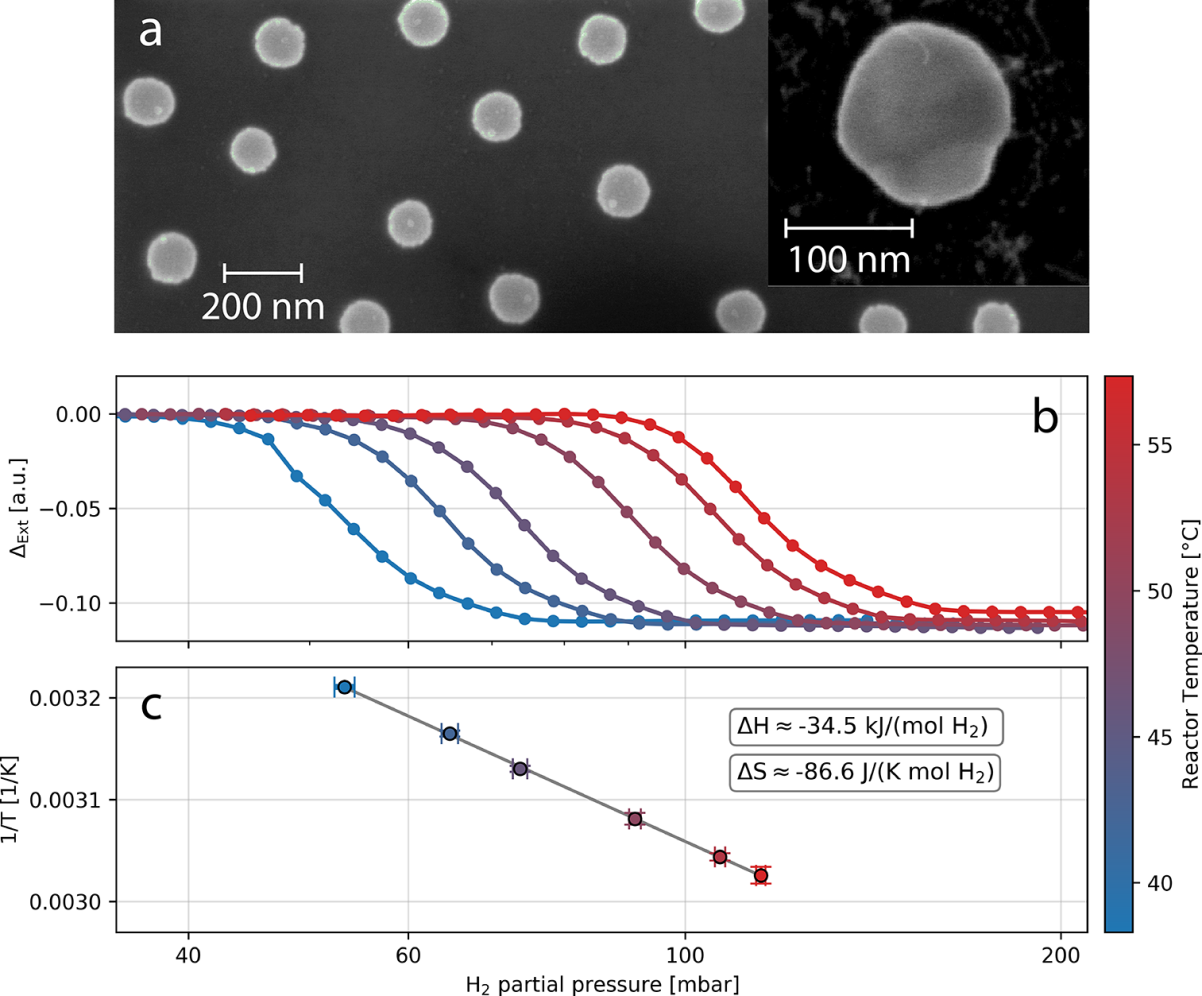
Constructing the Van’t Hoff calibration curve. (a) SEM image of an as-deposited Pd nanodisk quasi-random array sample fabricated with hole-mask-colloidal lithography. Inset: close-up image of an annealed Pd particle with thermally stable crystallinity. (b) Optical isotherms constructed from corresponding measurements at six different reactor temperatures (38.3, 42.8, 46.3, 51.4, 55.4, and 57.3 °C). (c) Inverse temperature plotted against the phase transition pressures extracted from the six isotherms in (b) resulting in a flipped Van’t Hoff plot. The extracted Δ*H* and Δ*S* values are in good agreement with the literature for the Pd–H system.^65−67^ This Van’t Hoff plot serves as the calibration curve to deduce the temperature of the Pd nanoparticles upon high-power illumination. The error bars along the *x*-axis represent the 95% confidence interval of the fit functions to define the phase transition pressure (for details see Figure S6). The error bars along the *y*-axis considers the deviation from the set temperature during one measurement cycle (*cf*. Figure S2b).

To demonstrate the hydrogen nanothermometry concept, we nanofabricated quasi-random arrays of Pd disks with a nominal diameter of 140 nm and a height of 25 nm on a 9 × 9 mm^2^ glass substrate using hole-mask-colloidal lithography,^62^ followed by a thermal annealing step to induce particle recrystallization and ensure a reproducible response upon subsequent hydrogenation (Figure 2a; see also Methods).^63^ The annealed sample was then placed in a quartz-tube plug-flow reactor inside a glass pocket holder with optical access^64^ and contacted with a spring-loaded thermocouple on its edge to monitor its global temperature (Figure S1). To enable measurements of optical isotherms at constant reactor temperature at different levels of illumination (and thus different local sample temperatures), the reactor was heated with constant power *via* a resistive heating coil; that is, no feed-back loop for temperature control was used. To compensate for this deliberate lack of active temperature control and ensure a stable reactor temperature during the experiments, we thermally equilibrated the system for 150 min prior to each measurement to reach a steady-state temperature value. During a measurement, we increased the hydrogen concentration in Ar carrier gas at a constant flow rate of 200 mL/min at atmospheric pressure. Simultaneously, we recorded self-referenced optical spectra across the 350–900 nm wavelength range, which means that we used a spectrum initially measured in pure Ar as the reference for all subsequently measured spectra.^61^ To construct an optical isotherm, we then extracted the difference in self-referenced optical extinction, , defined as the average extinction between 450 and 480 nm subtracted by the average extinction between 730 and 760 nm, and plot it *versus* hydrogen partial pressure (Figure 2b; see Figure S2 for raw data and Figure S3a for an explanation of the self-referencing approach).

To now construct a Van’t Hoff plot that serves as a “calibration curve” for unknown particle temperature determination *via* hydrogen nanothermometry, we measured optical isotherms at six different set reactor temperatures (Figure 2b and Figure S4), for which we used a low optical power (8.3 mW) halogen lamp to minimize any light-induced temperature increase. From these isotherms, we then extracted the phase transition pressure at each temperature^67,68^ (Figure S5) and plot them *versus* the inverse temperature on a semilogarithmic scale (Figure 2c). As a result, we can extract values of −34.5 ± 0.3 kJ/(mol H_2_) and −86.6 ± 0.9 J/(K mol H_2_) for the change in enthalpy, Δ*H*, and entropy, Δ*S*, respectively, which are in good agreement with the literature^65−67^ and thus validate our general procedure. Here we note that the phase transition (Figure 2b) is not an abrupt change but rather a gradual transition owing to the fact that the measurements are done on an array of Pd disks. As each particle has slightly different shapes, sizes, and grain structure, the phase transition happens at slightly different pressures, leading to a slanted transition for the ensemble.^67,69^

**Figure 3:**
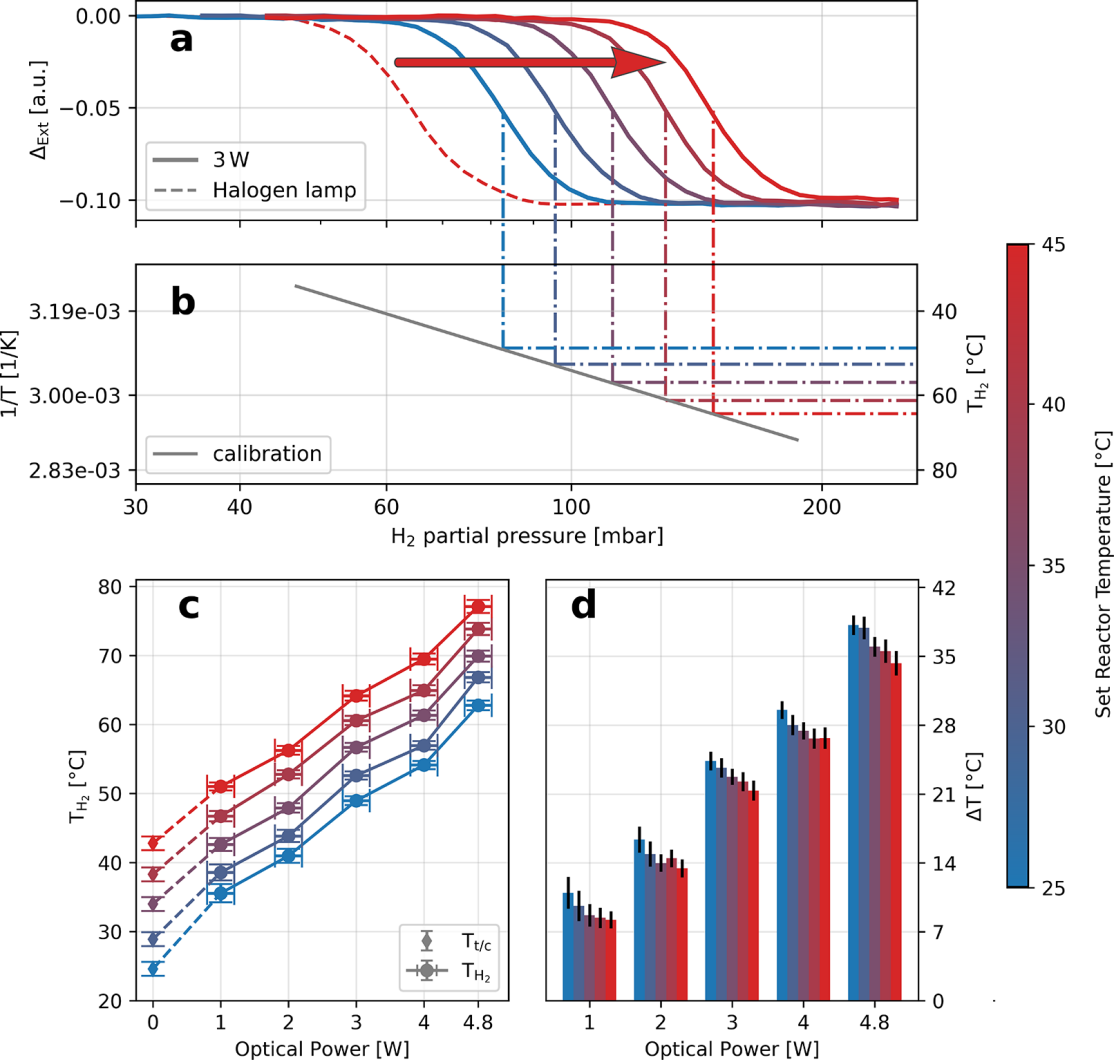
Determining the temperature increase in Pd nanoparticles upon illumination. (a) Representative isotherms measured at 3 W optical power for 24.6, 28.9, 34, 38.3, and 42.8 °C set reactor temperatures. A clear shift in the phase transition pressure compared to the calibration measurement with the halogen lamp (red arrow) is observed, indicating a significantly higher particle temperature. (b) Prerecorded calibration Van’t Hoff plot (gray line, *cf*. Figure 2c) used to determine the absolute particle temperature upon illumination with higher optical powers by determining the intersects of the phase transition pressures with the calibration curve. (c) Extracted absolute particle temperatures (*T*_H_2__) as a function of optical power for five different set reactor temperatures and the temperatures at 0 W (*T*_t/c_). A clear trend of increasing particle temperature with increasing optical power can be seen for all reactor temperatures. The error in *T*_H_2__ is derived from the 95% confidence interval in the linear fit to the Van’t Hoff calibration curve, and the error in the optical power is estimated to be ±0.2 W. (d) Temperature increase, Δ*T*, in Pd nanoparticles upon illumination as a function of optical power for the different set reactor temperatures. The values are obtained by subtracting the set reactor temperatures from *T*_H_2__. The error is obtained from the 95% confidence interval in the linear fit to the Van’t Hoff calibration curve combined with the standard deviation of the set reactor temperature measurement.

As the next step of our analysis, we set out to apply the calibrated response of our sample to determine the temperature increase induced by illumination with a high-power continuous wave plasma-arc lamp at different nominal sample temperatures. Specifically, we again measured optical isotherms but this time using the light from the plasma arc lamp for obtaining the self-referenced spectra (Figure S3b). To investigate the dependence of light-induced heating of the Pd particles on both set reactor temperature and optical power, we measured isotherms at five different set reactor temperatures (24.6, 28.9, 34, 38.3, and 42.8 °C) and at five different optical powers (1, 2, 3, 4, 4.8 W corresponding to 0.88, 1.77, 2.65, 3.54, and 4.24 W/cm^2^, respectively, measured outside of the reactor) obtained by defocusing the collimated beam (see Methods). Here we note that we below chose to discuss our results in terms of optical power, rather than power density, since the power density at the sample position is difficult to determine exactly due to the design of the reactor used and since the illuminated area remains constant in our experiments. Plotting the corresponding isotherms measured at five set reactor temperatures at 3 W reveals a distinct shift of the phase transition pressures compared to the reference measurement made with the low-power halogen lamp (Figure 3a). This upward shift indicates a significant temperature increase induced by illumination, which we can quantify by determining the intersection with the previously obtained calibration Van’t Hoff plot, resulting in the absolute nanoparticle temperature (*T*_H_2__, Figure 3b). Repeating this procedure systematically for the five set reactor temperatures and optical powers reveals a consistent and significantly higher temperature of the Pd nanoparticles upon illumination (Figure 3c), ranging from 35.5 °C at 1 W to 77.1 °C at 4.8 W. The data show a clear proportionality with an *R*^2^ > 0.98. By subtracting these temperatures with the corresponding set reactor temperatures, an absolute particle temperature increase, , of up to 38 °C is obtained upon high-power illumination (Figure 3d). These values agree well with the analytical model for the collective heating in plasmonic arrays developed by Baffou *etal*. (Figure S7).^70^ Furthermore, we notice a linear dependence of the absolute temperature increase on the set reactor temperature with an average *R*^2^ of 0.99 (Figure S8). We also note that the overall light absorption efficiency, *Q*_abs_, of the system in the pristine and hydride state changes by only ∼5%, as revealed by simulations (Figure S9). This finding means that the difference in light-induced heating when the system is in these two different phases is negligible for the overall interpretation of our results. Furthermore, we note that our approach can also be used to determine the optical power necessary to reach a specific particle temperature (see Figure S15 and corresponding discussion S1).

**Figure 4:**
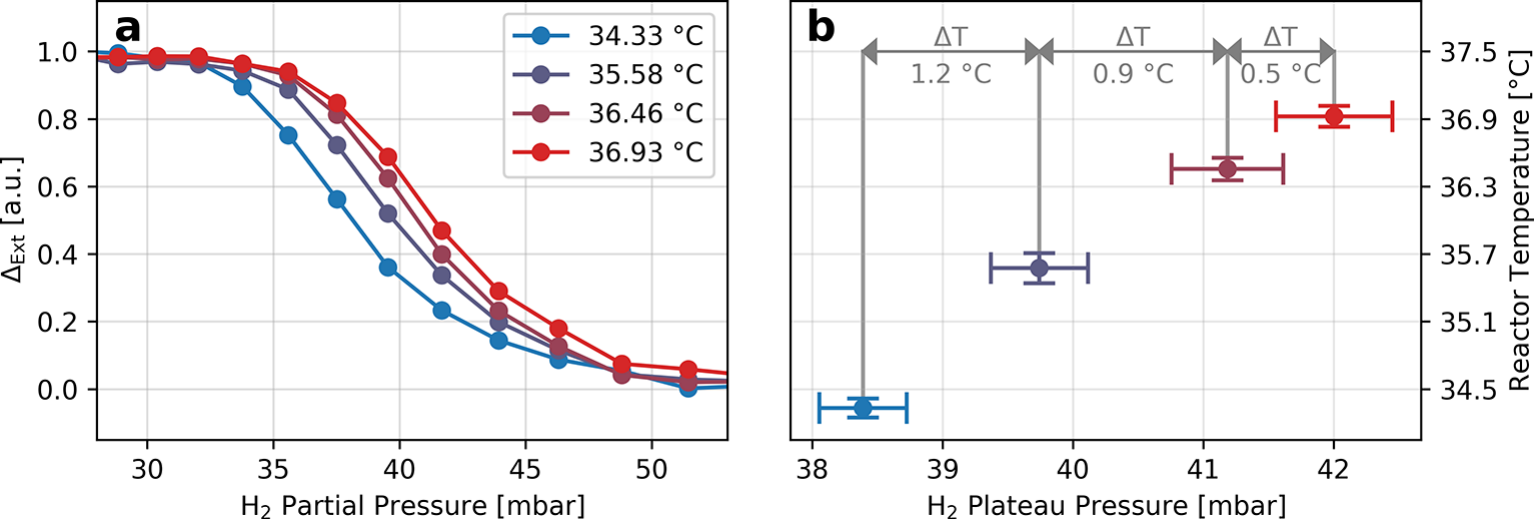
Deriving the temperature resolution of the hydrogen nanothermometry method. (a) Isotherms measured at four slightly different set reactor temperatures in a very narrow temperature range. (b) The extracted phase transition pressure is clearly distinguishable up to a temperature difference of 0.9 °C between isotherms. At a difference of 0.5 °C the pressure uncertainties (error bars defined by the combination of the standard errors of the fit functions) intersect and thus define a temperature resolution of 1 °C for the hydrogen thermometry in its present form. The error bars along the temperature axis are defined as the standard deviation of the temperature instability during one isotherm measurement cycle.

At this point it is also interesting to examine the temperature resolution of our hydrogen nanothermometry method. To do so, we conducted a series of isotherm measurements with deliberately small increasing temperature steps, to see if the method is able to distinguish such small temperature differences. Figure 4a shows the resulting isotherms measured at 34.33, 35.58, 36.46, and 36.93 °C, respectively. Clearly, the shift of the phase transition pressure in each isotherm is discernible, even for the two highest temperatures, which differ only by ∼0.5 °C. Quantitatively, however, when plotting the phase transition pressure of the isotherms obtained with the method explained above (Figure S6), their error confidence intervals overlap (Figure 4b). Looking at the next data points with a temperature interval of ∼0.9 °C, however, such an overlap does not exist. On the basis of this analysis, we thus conclude that our hydrogen nanothermometer exhibits a temperature resolution of 1 °C.

We also note that the final temperature resolution of a measurement is a trade-off against the speed of the measurement. If a wide temperature range is to be scanned to determine particle temperature with an accuracy of 1 °C, the measurement will take several hours, as each temperature step is on the order of a few minutes. However, by narrowing down the scanned temperature range or increasing the scanned pressure steps, the measurement can be sped up at the cost of temperature resolution. While this is sufficient for most applications, it is still significantly slower than fast photoluminescence methods that reach measurement rates of 80 kHz.^71^

**Figure 5:**
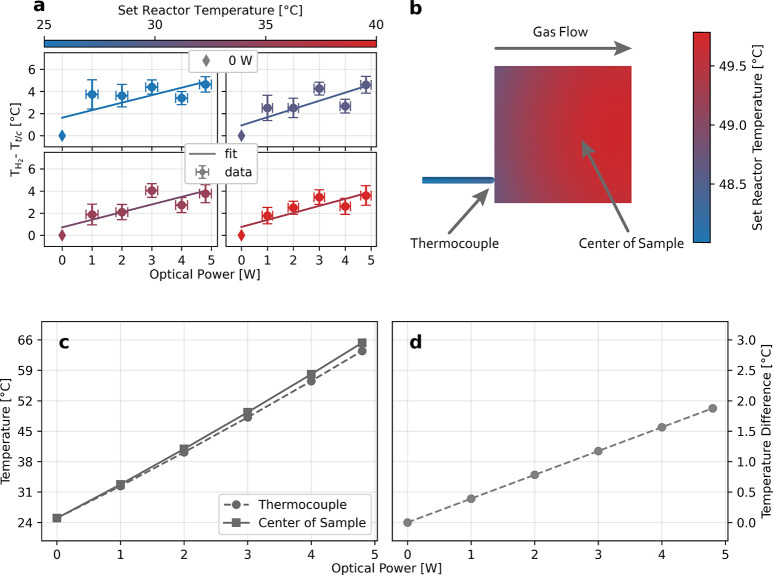
Temperature inhomogeneity on the sample and FDTD-informed conjugated heat transfer simulations of temperature distribution on the sample. (a) Temperature difference for a Pd nanoparticle array under illumination at different optical power and reactor temperatures, as obtained by comparing the temperature measured by a thermocouple (*T*_t/c_) that is touching the side of the sample and therefore represents the global sample temperature (including the substrate) and the temperature extracted by hydrogen nanothermometry (*T*_H_2__) that represents the absolute Pd particle temperature. A proportionality between increasing temperature difference and increasing optical power can be identified. The uncertainties along the *y*-axis are obtained by using the standard error of the linear fit function of the Van’t Hoff calibration curve and the uncertainty along the *x*-axis is estimated to be ±0.2 W. (b) Simulated temperature distribution across the sample and thermocouple mounted inside the reactor pocket (see Figure S18 for details of simulated geometries) for a set reactor temperature of 25 °C and a gas flow rate of 27 mL/min upon heating with an optical power of 3 W. (c) Extracted simulated temperature in the center of the sample and at the tip of the thermocouple plotted for 1, 2, 3, 4, and 4.8 W optical power. Notably, the discrepancy between the thermocouple and sample center temperature increases for increasing optical power. (d) Difference between thermocouple and sample center temperatures plotted as a function of irradiated optical power, revealing a proportionality, in reasonable agreement with the experiments (*cf*. Figure 5a).

Having established the ability of our approach to accurately measure the absolute temperature of Pd nanoparticles upon illumination with 1 °C resolution, it is now interesting to investigate whether the direct nature of our measurement also can reveal a temperature *difference* between the global sample temperature (*T*_t/c_) measured traditionally using a thermocouple positioned at the side of the sample (Figure S1b) and the real nanoparticle temperature in the sample center measured by our hydrogen nanothermometer (*T*_H_2__). For this purpose, we plot the difference between *T*_t/c_ and *T*_H_2__ as a function of irradiated power for four different set reactor temperatures (Figure 5a). As the main result, we find that *T*_H_2__ is universally higher than *T*_t/c_ obtained by the thermocouple. Furthermore, we identify a weak trend that this difference between *T*_H_2__ and *T*_t/c_ increases for higher optical power.

To put this result into perspective and to prepare grounds for a discussion of its implications in plasmonic applications, it is important to elucidate the origin of the observed effect. To this end, naïvely, one could argue that it is to be expected and the consequence of the Pd particles being hotter than the substrate because (i) light is mainly and efficiently absorbed in the particles^72^ and (ii) the thermocouple is in contact with the substrate, rather than with the particles themselves. While this is a tempting conclusion, calculations by Zhdanov and Kasemo for a geometrically very similar (quasi-2D) system actually predict that the temperature difference between a glass substrate and metal nanoparticles under illumination is almost negligible due to the high thermal conductivity of the system.^36^ These results have also been confirmed by Baffou *etal*.^35^ Therefore, a more careful analysis of the situation at hand in our experiments is necessary.

To do this, we start by identifying the geometry of our setup, as well as the fact that the measurements are carried out in plug-flow, as two likely factors of importance. Specifically, we note that the optical measurement of the isotherms used for temperature determination takes place in the center of a square sample where the light is irradiated with circular symmetry, whereas the thermocouple is positioned at the sample edge. This is important because to a first approximation it is reasonable to assume that the sample is heated homogeneously from the center since our nanofabrication produces a uniform distribution of the Pd nanoparticles on the glass substrate at the cm^2^ level.^62,73^ Hence, assuming first that there is no gas flow along the sample, this homogeneous heating combined with the square sample geometry will lead to the highest temperatures in the center of the samples. Consequently, a circular temperature gradient across the sample can be expected, with the hottest area in the middle and the coolest areas at the edges. Introducing a gas flow across the sample will slightly alter this situation due to forced convection that shifts the hot center downstream, with the amount of shift determined by the flow rate. This general hypothesis is indeed confirmed by conjugated heat transfer simulations using COMSOL Multiphysics using the Pd nanodisk light absorption calculated by the finite-difference time-domain (FDTD) method (Figure 5b; details about the simulations can be found in Figures S16–S19 and the corresponding discussion in the Supporting Information, SI, Section S2). The temperature gradient will also contribute to the fact that the phase transition from α- to β-phase is not abrupt but gradual, as each particle has a slightly different temperature and hence a slightly different phase transition pressure.

A second effect to consider is the presence of the thermocouple itself. As the first aspect, it has a small contact area with the sample and thus likely a relatively poor thermal contact, which may contribute to a lower temperature reading. As a second aspect, the thermocouple has a sizable thermal mass, which in combination with its placement upstream of the sample in the reactor and a concurrent cooling effect by the bypassing gas may further reduce the temperature reading compared to the sample temperature in the center. Again resorting to simulations, we can indeed confirm the proportionality between the increasing temperature difference of the thermocouple positioned at the center of the sample and optical power (Figure 5c,d), which is in reasonable agreement with the experiments (*cf*. Figure 5a).

**Figure 6:**
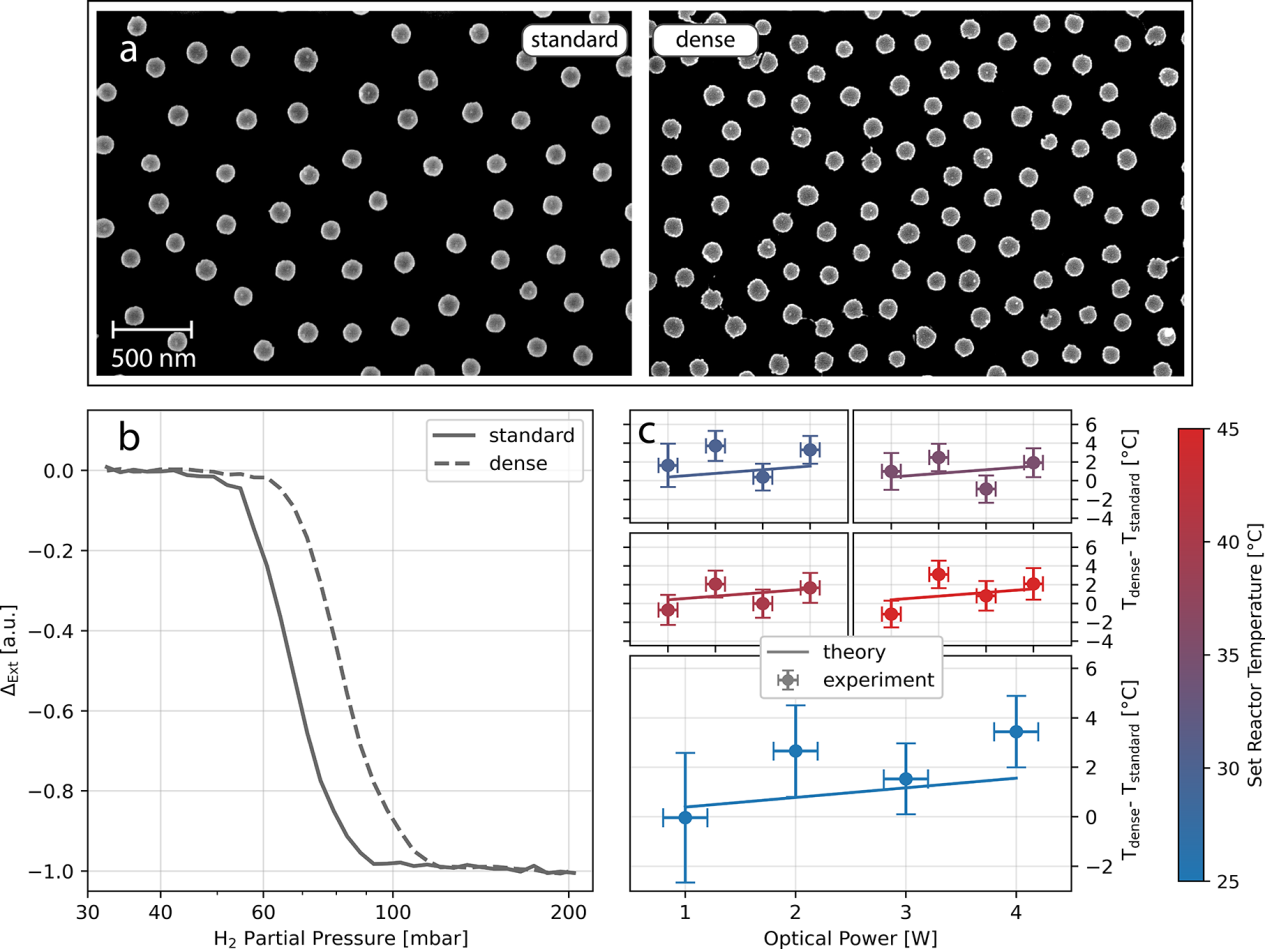
Measuring the light-induced temperature increase dependence on Pd nanoparticle array density. (a) SEM images of a standard Pd nanoparticle array with 11% surface coverage (left) and a dense Pd nanoparticle array with 18% surface coverage (right). (b) Optical isotherms of the two arrays obtained at identical 2 W optical power and 25 °C set reactor temperature. The denser array exhibits a higher phase transition pressure. Note that  is normalized to facilitate direct comparison between the two isotherms. (c) The measured and calculated (using eq 2; details in the SI Section S3) temperature difference between the standard and the dense sample obtained at 24.6, 28.9, 34, 38.3, and 42.8 °C set reactor temperature. For all optical powers the dense sample exhibits higher temperatures. The particles are corrected for ambient temperature to avoid the influence of slightly varying room temperature (raw data Figure S13). The depicted error in temperature difference is derived from the 95% confidence interval in the linear fit to the Van’t Hoff calibration curve, and the error in optical power is estimated to be ±0.2 W.

Having established and characterized in detail the hydrogen nanothermometry concept, we now put it to the test by nanofabricating a sample with higher Pd particle surface coverage compared to the standard sample (18% *vs* 11%, respectively; see Figure 6a). This increase in coverage is also associated with a decrease in the average interparticle distance from 331 to 250 nm, as determined from the corresponding radial distribution function (Figure S10).^74^ The idea behind this design is to demonstrate that an increased particle surface coverage leads to a larger temperature increase upon illumination due to a collective heating effect and that we are able to accurately measure this increase using hydrogen nanothermometry. Accordingly, we first compare optical isotherms of the dense and standard sample acquired at the same conditions and indeed find a significantly higher phase transition pressure for the surface with higher Pd particle coverage (Figure 6b). Translating these increased phase transition pressures (Figure S11) into a temperature increase induced by the higher Pd particle coverage, *T*_dense_ – *T*_standard_, we find a value of approximately 4 °C for 4 W optical power at 25 °C set reactor temperature, as well as the expected dependence of the temperature difference on irradiance (Figure 6c).

With this value in hand, it is now interesting to compare it to the result obtained when using the analytical expression for photoinduced heating in nanoparticle arrays established by Baffou *etal*.^70^ that suggests an inverse correlation between interparticle distance and heating upon illumination as
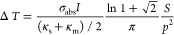
2where  is the nanoparticle absorption cross section, *I* is the irradiance,  and are the thermal conductivity of the substrate and the medium, respectively, *S* is the side length of the array, and *p* is the interparticle distance. To obtain  of a Pd nanodisk needed as input to estimate  for our system at hand, we employed FDTD simulations, which yield an average  = 1.32 × 10^–14^ m^2^. The remaining required input parameters used for the calculation, as well as further details, are summarized in the SI Section S3 and Methods sections. The accordingly calculated temperature increases upon illumination, , for two samples with a particle density difference as in our experiment are plotted in Figure S12. The temperature differences between these two samples are plotted Figure 6c, together with the corresponding experimental data points obtained at five different set reactor temperatures and for four irradiated powers. Notably, both absolute values and trends with respect to irradiated power exhibit good agreement. Furthermore, we obtained similar results using COMSOL simulations for a surface with 18% particle coverage (Figure S14). Altogether, these results thus validate the capability of hydrogen nanothermometry to accurately and directly measure the temperature of Pd nanoparticles upon illumination.

## Conclusions

In summary, we have presented a direct, noninvasive optical nanothermometry concept with 1 °C resolution that employs the temperature-sensitive first-order hydride formation in Pd nanoparticles as an intrinsic temperature probe. This use of the Pd nanoparticles themselves as the temperature reporter enables direct and absolute nanoparticle temperature measurements and overcomes limitations of traditional temperature probes that include large thermal mass or convolution of readout contributions that stem from the particles themselves and their support/surrounding medium, as well as temperature gradients on the sample. This was exemplified by identifying a difference between the temperature of a sample decorated with a quasi-random array of Pd nanoparticles measured using a thermocouple and the hydrogen nanothermometer, which arises due to a temperature gradient in the sample and limited heat transfer between sample and thermocouple. As a further aspect, applying the hydrogen thermometer, we were able to accurately measure the temperature difference between two surfaces with different Pd particle coverages—and thus different collective plasmon heating—at identical illumination conditions and corroborated these results by numerical simulations and a theoretical model. Consequently, the hydrogen nanothermometry developed here provides an alternative solution for accurate direct temperature measurements at the nanoscale, which we predict to find application in (plasmon-mediated) catalysis, where accurate temperature measurements of the catalyst are crucial and challenging to help distinguish between hot-carrier-mediated and photothermal reaction pathways. To this end, utilizing plasmonic nanoimaging hydrogen nanothermometry could be used to acquire thermal images with a spatial resolution down to 900 nm.^75^ Our method can also be useful in other applications that involve plasmonic nanoparticles that either are sensitive to temperature change or rely on deliberately induced temperature variations using the plasmonic effect. We also predict that the method potentially is capable of near to “real” time particle temperature determination when utilizing an experimental setup with low thermal mass (Figure S15). Finally, we also note that hydrogen nanothermometry is not restricted to Pd alone, since many other metals, such as niobium,^76^ yttrium,^77^ magnesium,^78^ or a wide range of metal alloys^58,79,80^ readily form hydrides and are also plasmonically active.

## Methods

### Nanofabrication

The samples were nanofabricated using the hole-mask colloidal lithography (HCL) technique.^62^ For the isotherm measurements the samples were fabricated on fused silica substrates and for SEM imaging on an oxidized silicon wafer. The following instruments were used during the nanofabrication: spin-coating (Suss, LabSpin6), oxygen ion etching (Plasmatherm, BatchTop m/95), and electron beam evaporation (Lesker, PVD 225). To define the mask in the fabrication process, we used a 0.2 wt % 140 nm diameter sulfate latex bead solution (molecular probes by Life Technologies) in deionized water. To create a surface with higher particle density, we added 0.1 mmol/L NaCl to the sulfate latex bead solution to screen the surface charges of the polystyrene beads and thereby achieve a higher density array upon their self-assembly.

### Experimental Setup

The measurements were executed in a quartz-tube plug-flow-type reactor (X1, Insplorion AB, Göteborg, Sweden) enclosed by a heating coil, thermal isolation, and a metal shield. The sample was mounted in a custom optically transparent pocket reactor^81^ with a spring-loaded thermocouple touching the side of the sample. The gas flow through the reactor was controlled by an array of mass flow controllers (Bronkhorst Low-ΔP). To ensure a constant pressure inside the reactor, an upstream pressure controller was used (Bronkhorst El-Press P-702CV). The reactor was also equipped with optical access to allow for transmission measurements using a fiber-coupled spectrometer (Avantes AvaSpec-1024) and halogen lamp (Avantes Avalight-HAL-B), high-power halogen light source (250 W halogen lamp in a Newport 67011 QTH housing without optical IR filter), or a mercury xenon arc light source (Newport 6295NS in a 66921 housing and a 6123NS liquid optical IR filter). The optical power of the mercury xenon arc light source was measured with a digital optical power meter (Thorlabs PM100D) connected to thermal power sensors (Thorlabs S425C-L and S470C) directly connected to the output of the lamp. H_2_ (4% ± 2 rel % diluted in Ar carrier gas) and Ar carrier gas (99.9999% purity) were used. Prior to the first isotherm measurement the samples were annealed at 500 °C in 4% H_2_ with a flow of 200 mL/min for 12 h to induce grain growth and thereby induce reproducible response upon H_2_ exposure.^63^

### Scanning Electron Microscopy

The SEM images were recorded with the in-lens system of a Zeiss Supra 55 VP. The acceleration voltage was 5 kV, and the working distance was at least 5 mm.

### FDTD Simulations

The FDTD simulations were conducted with Ansys-Lumericals FDTD Solution version 8.26.2717 to calculate the absorbed power and absorption cross section of a Pd nanodisk. The simulated geometry was a Pd disk with a 140 nm diameter and a height of 25 nm placed on a SiO_2_ substrate. The dielectric functions for Pd and SiO_2_ were used out of Ansys-Lumerical’s database originating from Palik *etal*.^82^ The source was a total-field/scattered field source with a linearly polarized plane wave.

### Heat Distribution Simulations

To simulate the heat distribution in the system, COMSOL Multiphysics 5.6 with a conjugated heat transfer setup was used. The 9 × 9 × 0.5 mm^3^ fused silica substrate used in the experiment was modeled in a glass pocket of 25 × 12 × 1 mm^3^, with a wall thickness of 1 mm, to mimic the reactor pocket. The inside of the pocket was filled with air. The thermocouple, touching the side of the sample, was made of Inconel 600 with a diameter of 0.5 mm. The material constants were taken from the COMSOL Multiphysics database. The flow rate through the pocket was 27 mL/min and adjusted to match the temperature in the experiments at 3 W optical power and 25 °C set reactor temperature within ±1 °C. The details of the heat distribution simulations are explained in the Supporting Information (Section S2).

## Supporting Information Available

Schematic of experimental setup, representative segments of the measured extinction difference under increasing hydrogen pressure, self- and substrate-referenced spectra in Ar and H_2_, change in extinction difference under increasing H_2_ partial pressure at 3 W and 45 °C, determination of H_2_ phase transition pressure, determination of the H_2_ phase transition pressure uncertainty, temperature increase upon illumination compared to an analytical model for photoinduced heating, Pd particle temperature at different reactor temperatures and optical powers, calculated absorption efficiency for Pd and Pd-hydride nanodisks, radial distribution functions of the standard and dense sample, optical isotherms and Van’t Hoff plot of a dense sample measured with a halogen lamp, calculated temperature increase upon illumination for standard and dense sample, Pd particle temperature for standard and dense sample at different optical powers and reactor temperatures, COMSOL calculated temperature differences of standard and dense sample at different optical powers and reactor temperatures, Van’t Hoff calibration and optical isotherm obtained by varying the power density, FDTD calculated absorbed power and absorption cross section for single Pd disk, temperature increase upon illumination for an empty glass substrate and a glass substrate with a Pd nanoparticle array, modeled experimental setup for COMSOL calculations, COMSOL calculated temperatures at the center and edge of the sample for different gas flow rates (PDF)
