# Supplementary material for: Optical Hydrogen Nanothermometry of Plasmonic Nanoparticles under Illumination
Source: ACS Nano. 2022 Mar 28;16(4):6233–43. doi: 10.1021/acsnano.2c00035 (PMC9047005; doi:10.1021/acsnano.2c00035)
Supplement: Supplementary file 1 — nn2c00035_si_001.pdf [file nn2c00035_si_001.pdf]

Supporting Information

for

Optical Hydrogen Nanothermometry of Plasmonic Nanoparticles Under Illumination

Christopher Tiburski[1], *Ferry Anggoro Ardy Nugroho*[2,*], *Christoph Langhammer*[1,*]

[1]Department of Physics, Chalmers University of Technology, 412 96 Göteborg, Sweden

[2]Department of Physics and Astronomy, Vrije Universiteit Amsterdam, De Boelelaan 1081, 1081 HV Amsterdam, The Netherlands

* Correspondence to: ferryanggoroardynugroho@yahoo.com; clangham@chalmers.se

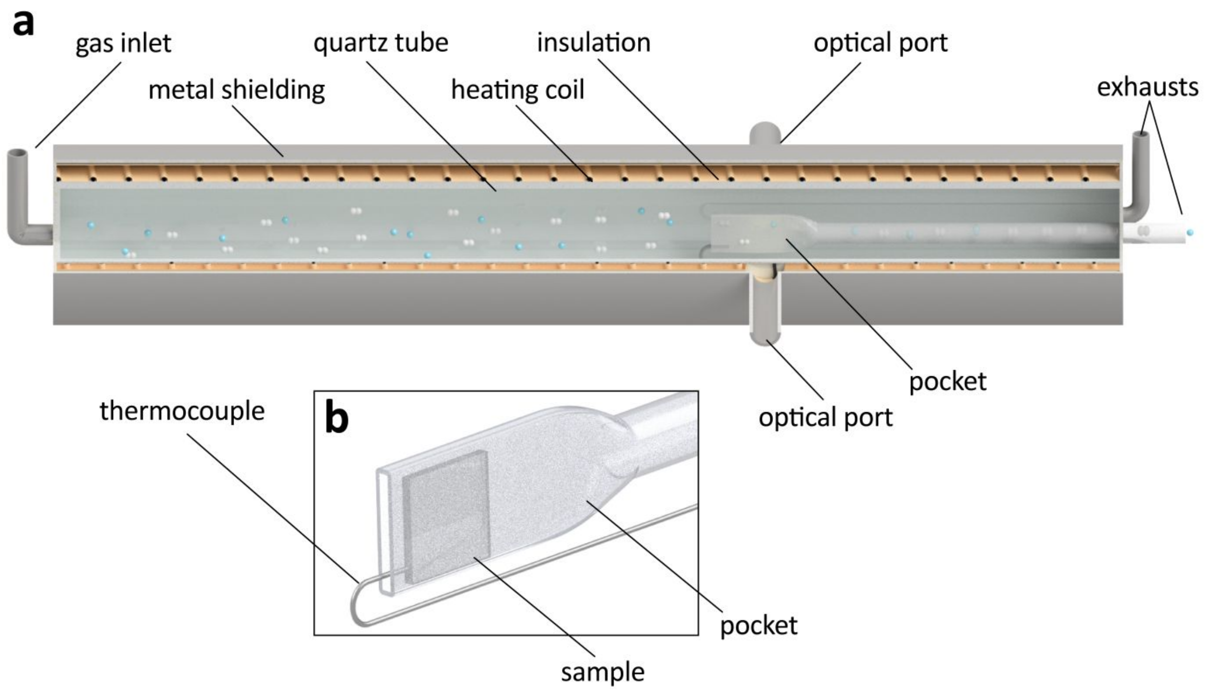

Figure S1: *a) Schematic of the plug-flow reactor composed of a quartz tube with external shielding and resistive heating. The optical ports allow for illumination and in situ spectroscopic measurements. The gas composition is controlled by an array of mass flow controllers on the inlet side. b) The sample is placed inside a glass pocket mounted inside the quartz tube and a spring-loaded thermocouple is monitoring global sample temperature.*

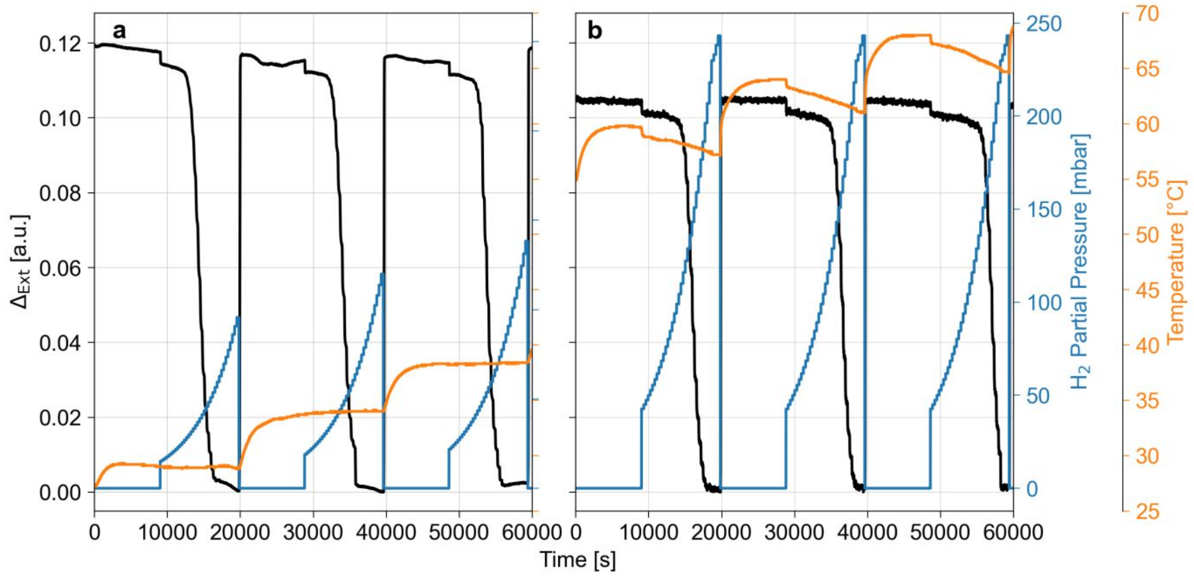

Figure S2: *A representative segment of the measured extinction difference,* Δ_{Ext}*, of Pd nanoparticles under step-wise increasing hydrogen partial pressure at different set reactor temperatures upon illumination of (a) a halogen lamp at 8.3 mW optical power and of (b) a plasma-arc lamp at 4.8W optical power. The drop of temperature in (b) arises due to the increased heat capacity of the gas mixture with increased hydrogen concentration.*[1] *The* Δ_{Ext} *and* H_2 *partial pressure data used to create a Van 't Hoff plot are described in* ***Figure S4****.*

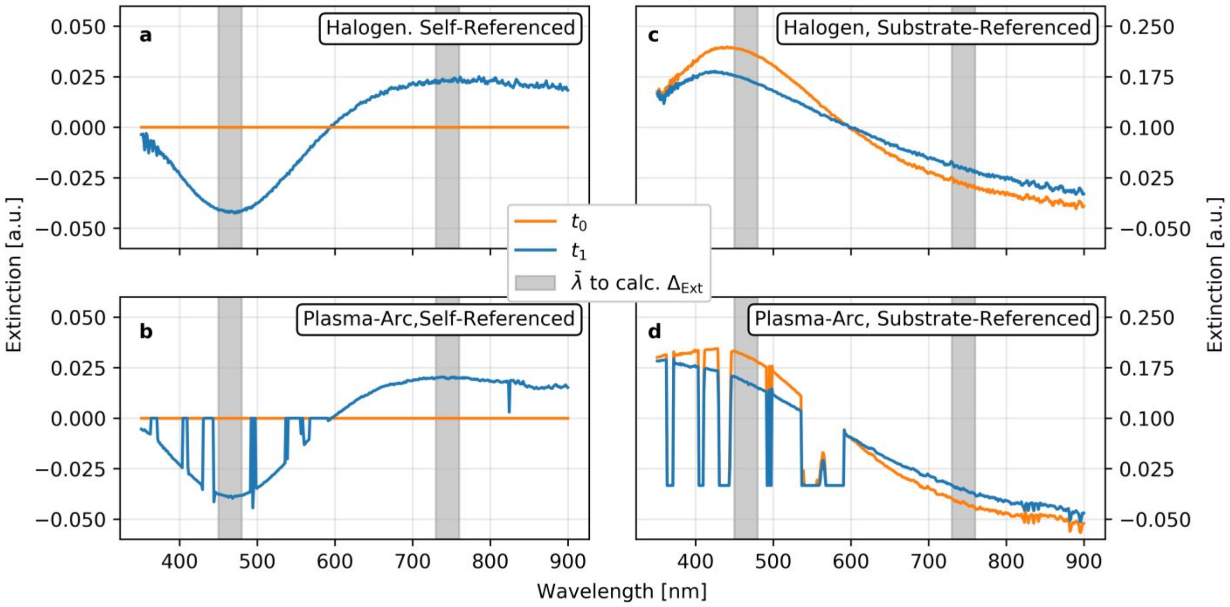

Figure S3: *a, b) Self-referenced initial extinction spectrum in Ar taken at the beginning of the measurement (t_0) plotted together with a representative spectrum at high hydrogen partial pressure in the reactor at t_1. We note the appearance of a maximum and minimum at long and short wavelengths, respectively, which appear as a consequence of hydrogen sorption and the corresponding shift and the change in extinction of the LSPR peak as can be seen in the substrate-referenced spectra (c, d). These data were measured using (a, c) the low-power halogen light source and (b, d) the high-power plasma-arc lamp. In (b, d), the spectrum at t_1 exhibits a region of apparent zero extinction as a consequence of high intensity emission peaks that saturate the CCD pixels of the fixed-grating spectrometer at these wavelengths. The grey areas denote the wavelength ranges used to calculate the extinction difference in the figures in the main text. They are selected such that they (i) are close to the maxima of the spectral features induced by hydrogen sorption and (ii) do not overlap with areas where pixel saturation occurs in experiments using the plasma-arc lamp.*

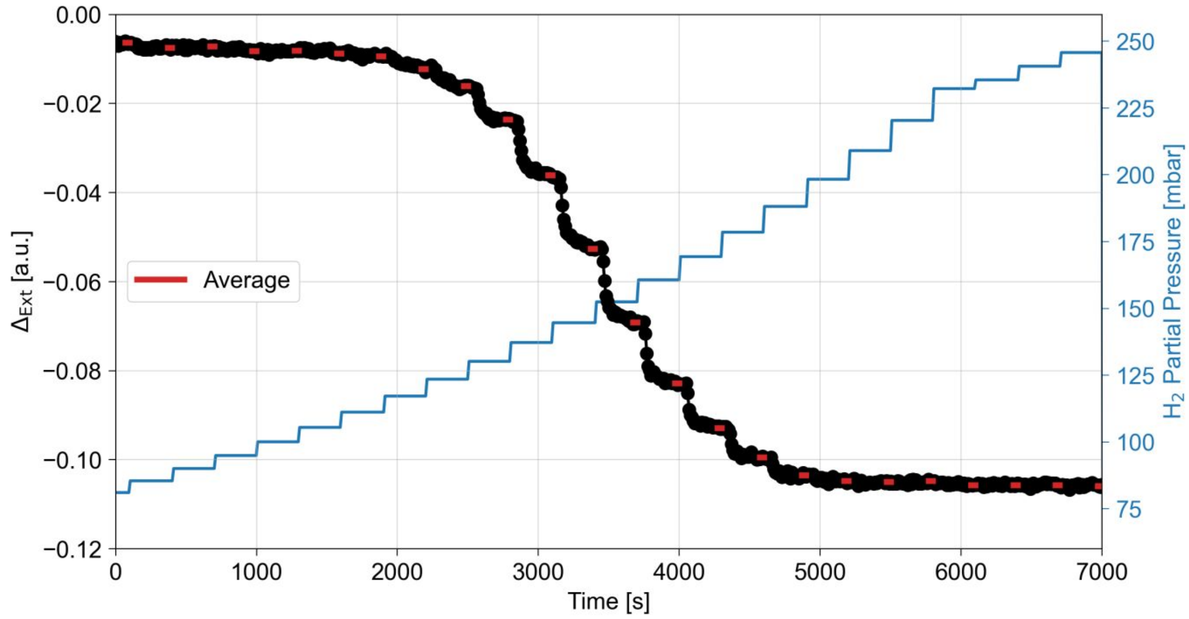

Figure S4: *The Δ_{Ext} induced by increasing hydrogen partial pressure measured at 3 W optical power and 45 °C set reactor temperature. The Δ_{Ext} data corresponding to the last one tenth of each H_2 partial pressure step (marked in red) are averaged and used to construct the isotherm.*

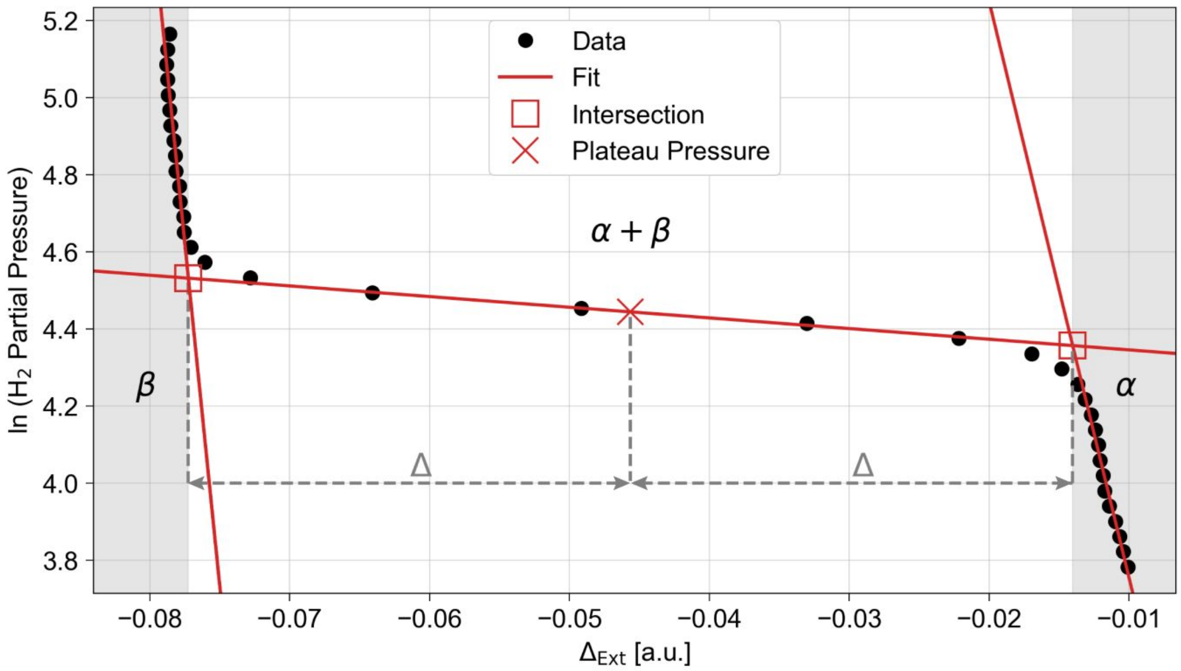

Figure S5: *To determine the phase transition pressure, linear fits are applied to the three distinct isotherm regions (i.e., α-, β-, and α+β phases). The phase transition pressure is then defined as the midpoint of the two intersections, as indicated by the red cross.*

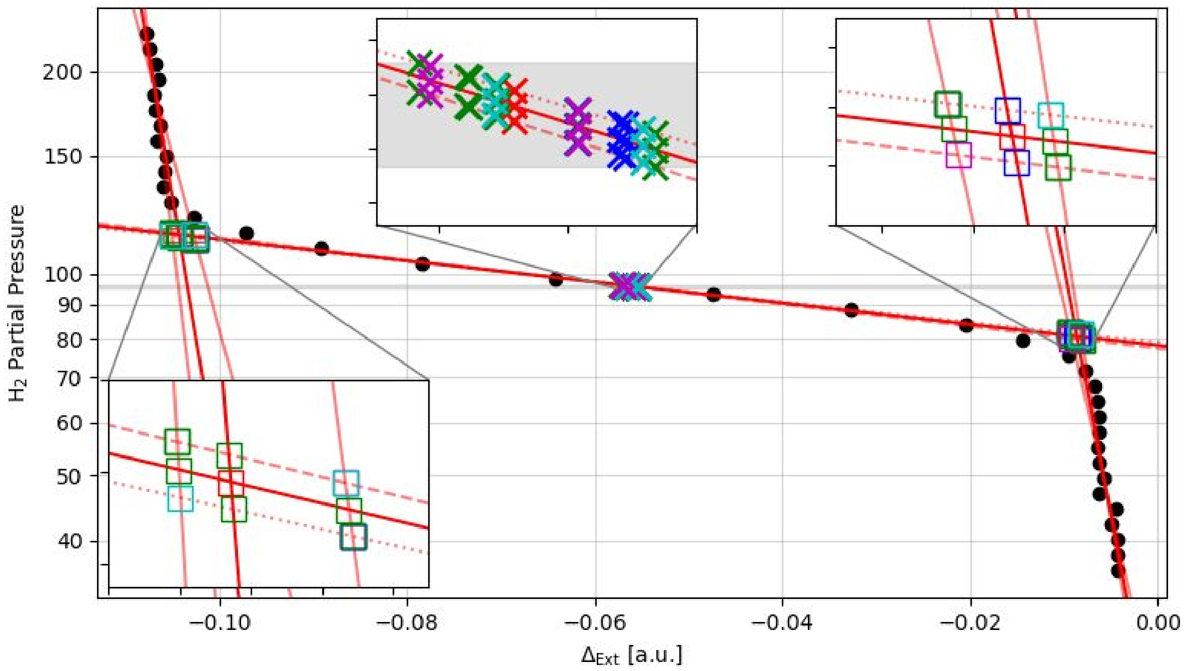

Figure S6: *To define the uncertainty in determining the phase transition pressure, we calculated all possible intersections of the fit functions of the three regions within their 95% confidence interval. The uncertainty range is then defined as the region between the highest and lowest pressure (marked in grey) of all possible phase transition pressures.*

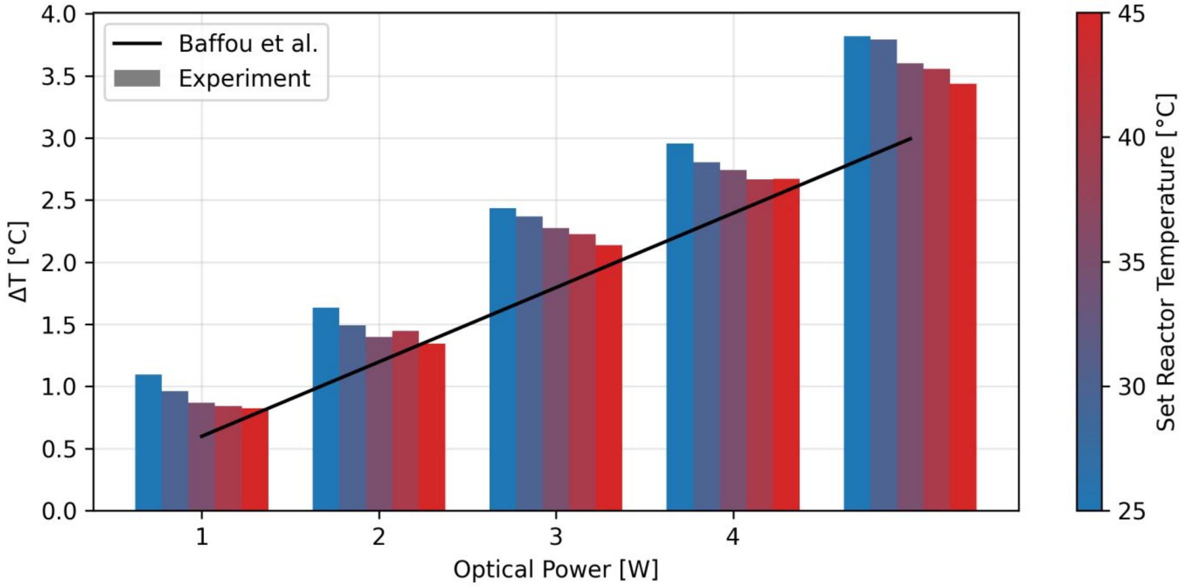

Figure S7: *Temperature increase, ΔT, in Pd nanoparticles upon illumination as a function of optical power for the different set reactor temperatures plotted together with the analytical model for photoinduced heating in nanoparticle arrays established by Baffou et al.*[2] *Evidently, the experimental values agree well with the model. The experimental values are scaled to 10% of the original values as only around 10% of the temperature increase comes from plasmonic heating as discussed in* ***Section S2*** *and* ***Figure S1****7. The analytical model is discussed in detail in* ***Section S3****.*

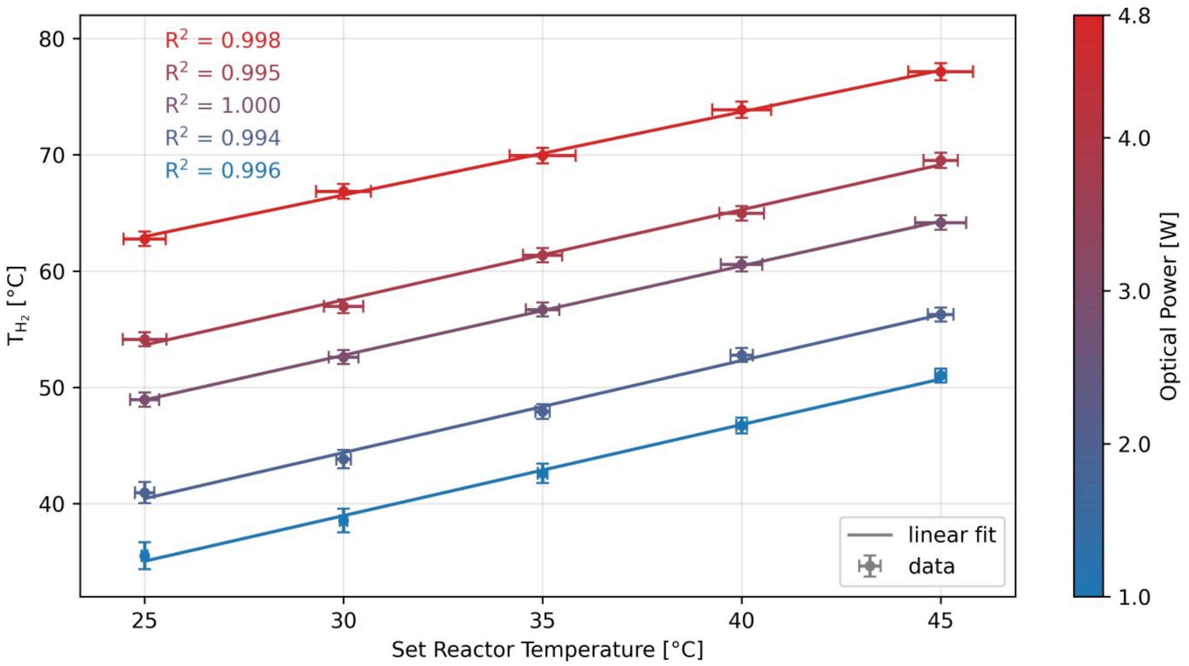

Figure S8: *Pd nanoparticle temperatures extracted by hydrogen nanothermometry for 1, 2, 3, 4, and 4.8 W optical powers vs the set reactor temperature. A clear linear trend (see* R^2*) of increasing nanoparticle temperature with increasing optical power for all reactor temperatures is observed. The error in hydrogen temperature corresponds to the 95% confidence interval of the linear fit function to the Van 't Hoff calibration curve and the error in the set reactor temperature is the standard deviation of the temperature during a measurement step.*

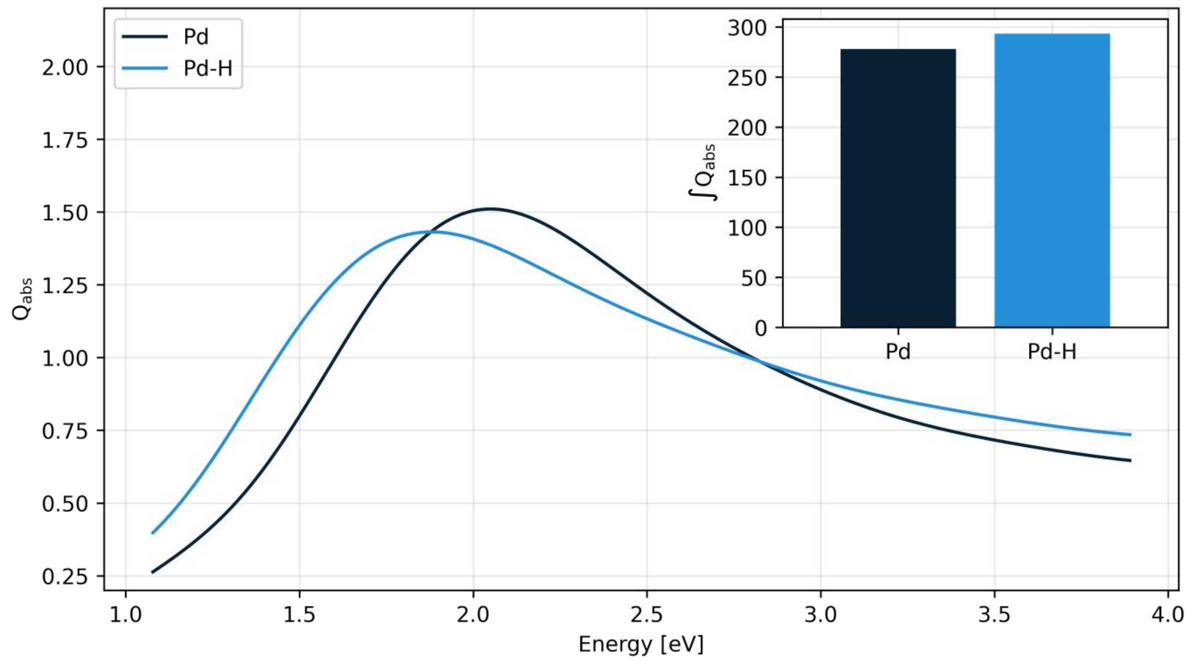

Figure S9: *The absorption efficiency, Q_{abs}, for a Pd and Pd-hydride nanodisk with 140 nm diameter and 25 nm height calculated with FDTD. The area $\int Q_{abs}$ between the pristine and hydride state varies only with ~5%. The dielectric functions are taken from Palm et al.* [3]

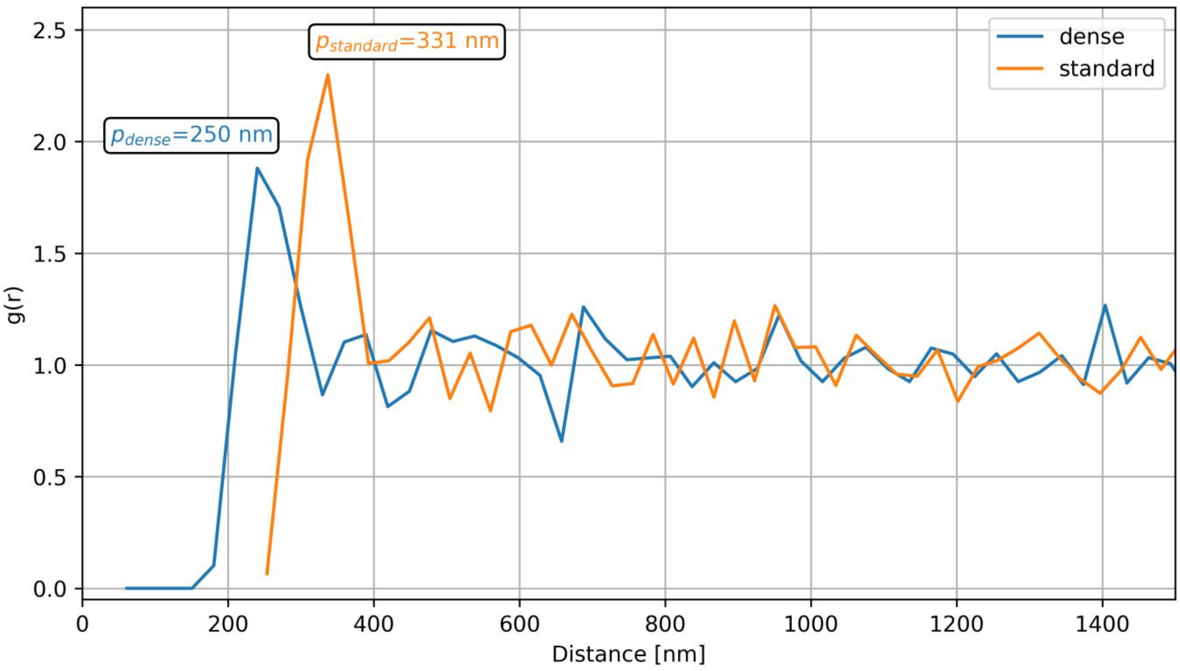

Figure S10: *The radial distribution function (RDF), g(r), of the standard and dense samples extracted from the corresponding SEM image (**Figure 6a**). The primary peaks indicate an average interparticle distance of $p_{standard}$ = 250 nm and p_{dense} = 331 nm for the standard and dense sample, respectively.*

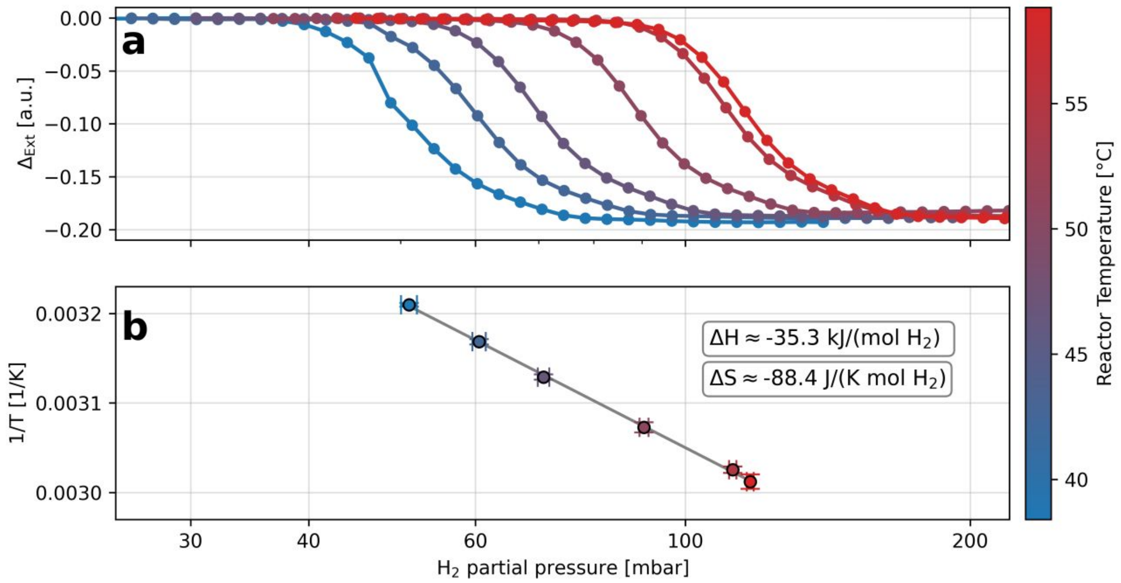

Figure S11: *a) Optical isotherms of the dense sample with 18% Pd particle surface coverage. The isotherms are constructed from corresponding measurements at six different reactor temperatures. b) The inverse temperature plotted against the phase transition pressures extracted from the six isotherms in (a) resulting in a flipped Van 't Hoff plot. The extracted ΔH and ΔS values are in good agreement with the literature for the Pd-H* system. *The error along the x-axis represents the 95% confidence interval of the fit functions to define the phase transition pressure (for details see* ***Figure S6****). The error along the y-axis considers the deviation from the set temperature during one measurement cycle (cf.* ***Figure S2b****).*

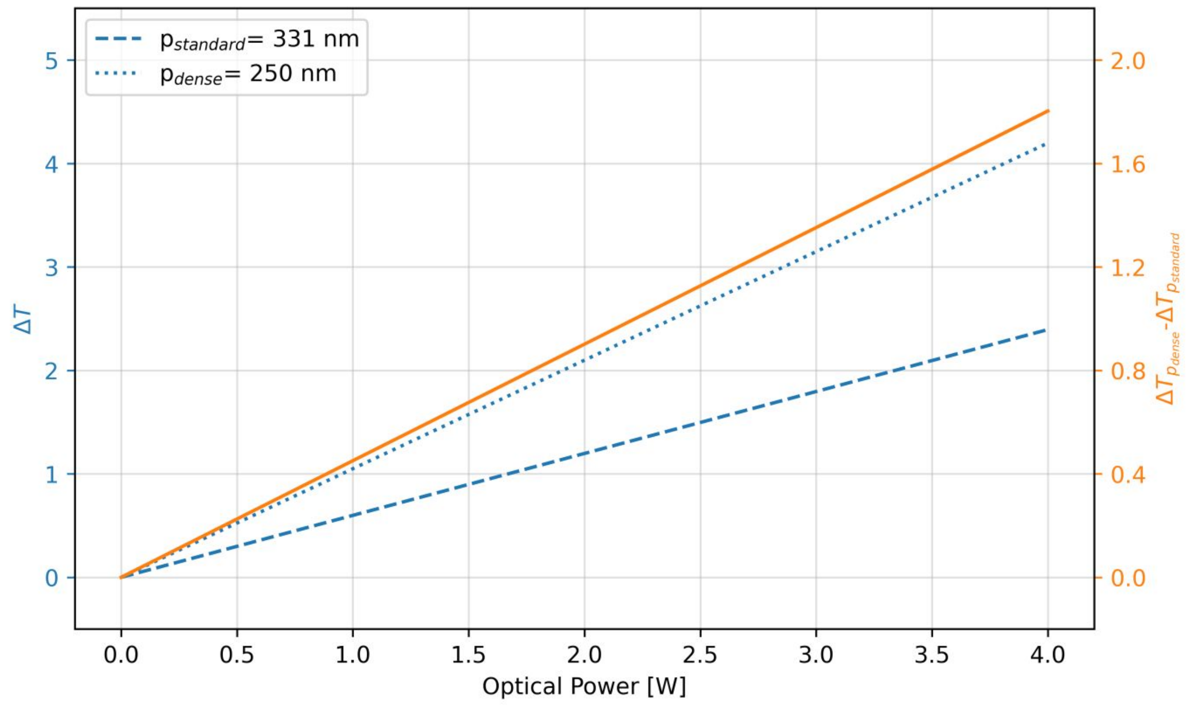

Figure S12: *The temperature increase, ΔT, due to photothermal heating of a Pd nanoparticle array at different optical powers for two samples with different interparticle distances, p, calculated with Eq. S3. The dense array with* p_{dense} *= 250 nm consistently shows a higher increase in temperature compared to the standard array with* $p_{standard}$ *= 331 nm. The details of the calculation are discussed in SI Section S2.*

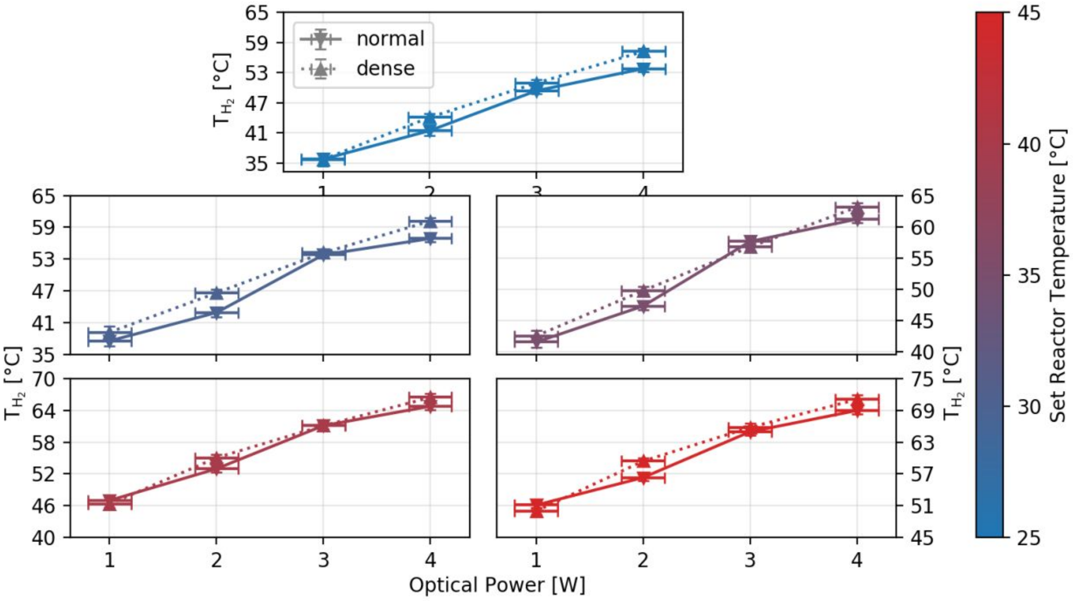

Figure S13: *The temperatures of the standard and dense sample measured by hydrogen nanothermometry for different optical powers and at different set reactor temperatures. As the measured particle temperature is highly sensitive to the temperature in the room where the measurement was conducted the data was adjusted accordingly to compensate for fluctuations in the room temperature. For example, if the data was recorded at 0.5 °C lower room temperature than the standard of 22 °C, the data were shifted by 0.5 °C to correct for this fluctuation. The dense sample shows a slightly higher temperature, as expected. The error in* $T^{H}{}_2$ *is derived from the 95% confidence interval in the linear fit to the Van 't Hoff calibration curve plus an estimated error of 0.2 °C for the room temperature measurement. The error in the optical power is estimated to be ±0.2 W.*

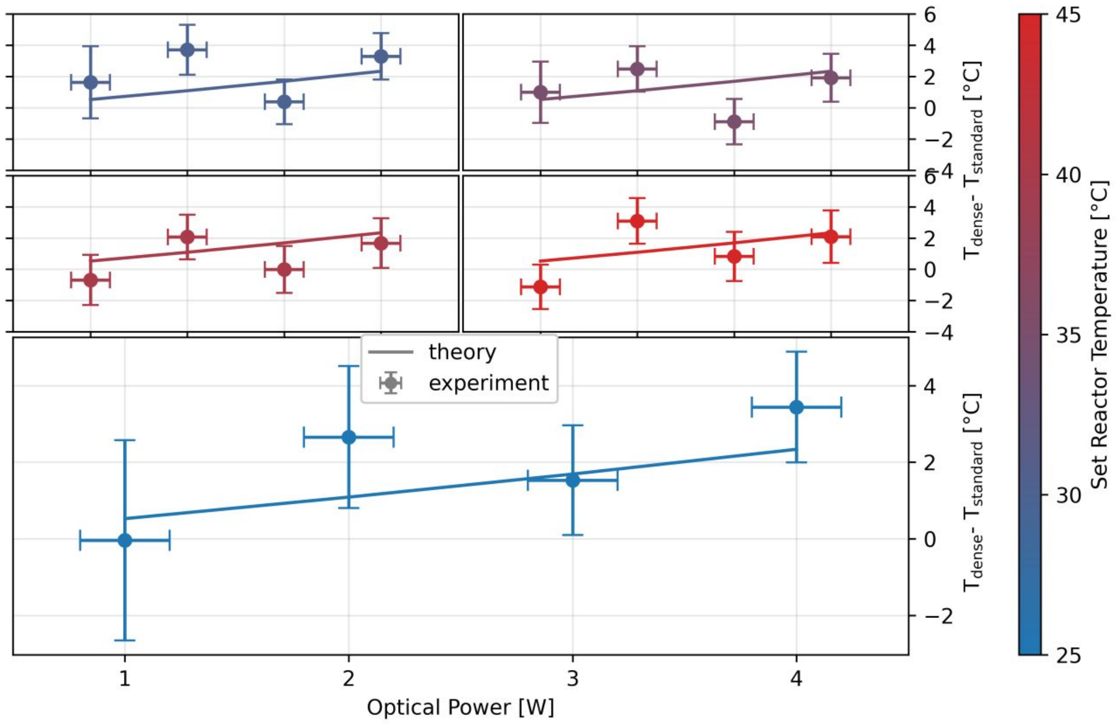

Figure S14: *The measured and calculated temperature difference of the standard and the dense sample at 24.6, 28.9, 34, 38.3, and 42.8 °C set reactor temperature. The calculations were done with COMSOL for a Pd particle surface coverage of 11% and 18 %, as described in the SI section S1. The experimentally measured particle temperatures are corrected for fluctuations of the ambient temperature in the room, as was described in* ***Figure S13****. The error in T_{dense} and $T_{standard}$ is derived from the 95% confidence interval in the linear fit to the Van 't Hoff calibration curve plus an estimated error of 0.2 °C for the room temperature measurement. The error in the optical power is estimated to be ±0.2 W.*

Section S1: Critical Optical Power for Specific Sample Temperature

Our approach cannot just be used to measure particle temperature by varying the hydrogen pressure but also to determine the optical power necessary to reach a specific sample temperature. To demonstrate this concept in a proof-of-principle fashion, we set out to determine the optical power of a halogen light source that is required to reach an absolute Pd nanoparticle temperature of 50 °C. In a first step, we determined the hydrogen pressure required at 50 °C to induce the α-β phase transition by comparing to the pre-recorded Van 't Hoff plot, which results in a critical pressure of 81.4 mbar (**Figure S15a**). In the next step, we measured optical extinction spectra from the sample at a constant H_2 pressure of 81.4 mbar, while varying the output from 6.5 W to 2.3 W in eight steps. After data processing with the self-referencing approach for each power step (*cf.* **Figure S3**), we then end up with an isotherm with the optical power on the x-axis (**Figure S15b**), from which the required optical power to reach 50 °C particle temperature can be deduced.

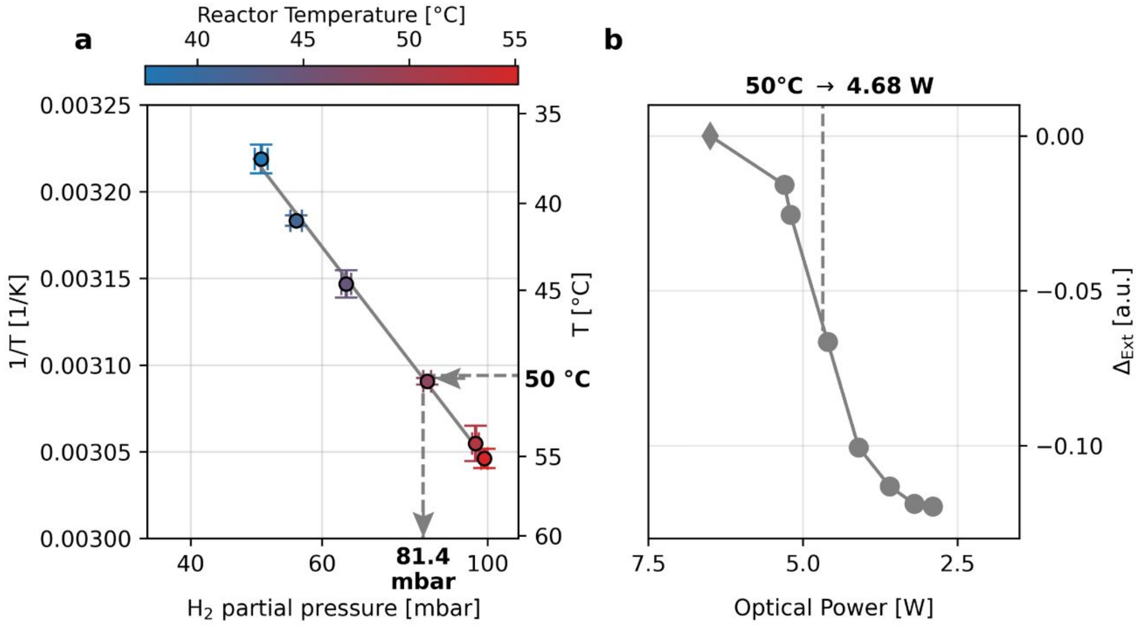

Figure S15: *a) A Van 't Hoff calibration plot used to find a critical optical power needed to reach a particular absolute particle temperature upon illumination. In this case, a Pd nanoparticle is 50 °C when it exhibits the α-β transformation plateau at 81.4 mbar H_2 partial pressure (gray arrows). b) Using this information, when exposed to constant H_2 pressure of 81.4 mbar and varying illumination power (from 6.5 W to 2.3 W), the self-reference spectra of the nanoparticles (cf.* **Figure S3***) exhibits a typical optical isotherm. By determining the optical*

power at which the phase transition happens one obtains the critical power to reach the 50 °C particle temperature that is 4.68 W. Note that data of 6.5 W was obtained in a separate measurement, as the setup did not allow for power changes of the needed magnitude in one measurement and was therefore marked separately (♦).

Section S2: Heat Distribution Simulations

To simulate the heat distribution on our samples we first calculated the absorbed power by the sample, using an FDTD simulation of the absorption cross section of a single Pd disk with a diameter of 140 nm of and a height of 25 nm as key input. The power absorption was normalized to the source power (see **Figure S16**) and the absorption of the glass substrate was neglected. As the total absorbed power, we used the integrated absorption over the visible light range which is $P_{abs} \approx 3.7\%$ of the source power. This assumption is reasonable because the plasma-arc light source used in the experiment is equipped with an IR filter and because the light has to pass multiple glass surfaces which remove most of the UV light before reaching the sample.

To get an estimate for a general temperature increase of the pocket reactor holding the sample induced by illumination, we measured this temperature increase with a thermocouple by illuminating a blank glass substrate inside the reactor and compared it to the results of a substrate with nanoparticles (**Figure S17**). This comparison shows that the temperature is only few degrees higher with particles on the substrate, and that the heating due to the particles only accounts for around 10% of the total temperature increase. Therefore, we introduced a parameter, $f = 0.1$, to in the calculation quantify how much power is absorbed by the sample and how much by the setup.

Furthermore, the amount of power absorbed by the sample also depends on the coverage of the nanoparticles. Therefore, we took SEM images of the different samples (**Figure 6a**) and determined the Pd particle surface coverage, θ, obtaining $\theta = 0.11$ and $\theta = 0.18$ for the standard and dense sample, respectively.

All the above-mentioned factors were summarized in a pre-factor to calculate the power absorbed by the samples depending on the lamp power output. The pre-factor, P_f, is defined as

$$P_f = P_{\text{abs}}\,\theta\,f \qquad \text{(S1)}$$

where P_{abs} is the mean absorption by the particles, θ the particle surface coverage of the sample, and f the fraction of light that reaches the sample. For the case at hand, we calculated the pre-factor, $P_f = 4.07 \cdot 10^{-4}$ using the following input values: P_{abs}= 0.037 , θ = 0.11, f= 0.10.

To simulate the heat distribution, we used COMSOL Multiphysics 5.6 with a conjugated heat transfer setup. The 9×9×0.5 mm^3 glass substrate was modeled in a glass pocket of 25×12×1 mm^3 with 1 mm wall thickness to also include the reactor pocket in the simulation. The pocket reactor is filled with air and a thermocouple made of Inconel 600 with a diameter of 0.5 mm touching the side of the sample is introduced (**Figure S18**). The material constants were taken from the COMSOL Multiphysics database.

The heat sources were the sample itself with a heating rate of

$$P = P_f \, P_{\mathrm{lamp}} \qquad \text{(S2)}$$

where P_{lamp} is the optical power of the lamp that is varied between 1 to 4.8 W. The pocket reactor is as a second heat source representing the power absorbed by the reactor with a heating rate such that the power absorbed by the pocket is 10% of the total absorbed power.

The last parameter of the simulation, the gas flow rate through the reactor pocket, was set to 27 ml/min. This value was chosen to closely match the temperature increase we see during the measurement. Thereby, the flow rate in the simulation was around 8 times higher than the real one in the experiment. This choice is also further justified because in the real setup there are more pathways for cooling, with the biggest contribution stemming from the outside of the pocket by the gas streaming by on the outside of the pocket (inside the reactor tube – c.f. Fig SI1). Therefore, it is natural that the *only* cooling path in the simulation needs to be increased to match the experimental results. More importantly, we show that the flow rate has only a minor influence on the temperature difference between the center of the sample and the thermocouple – see **Figure S19** below.

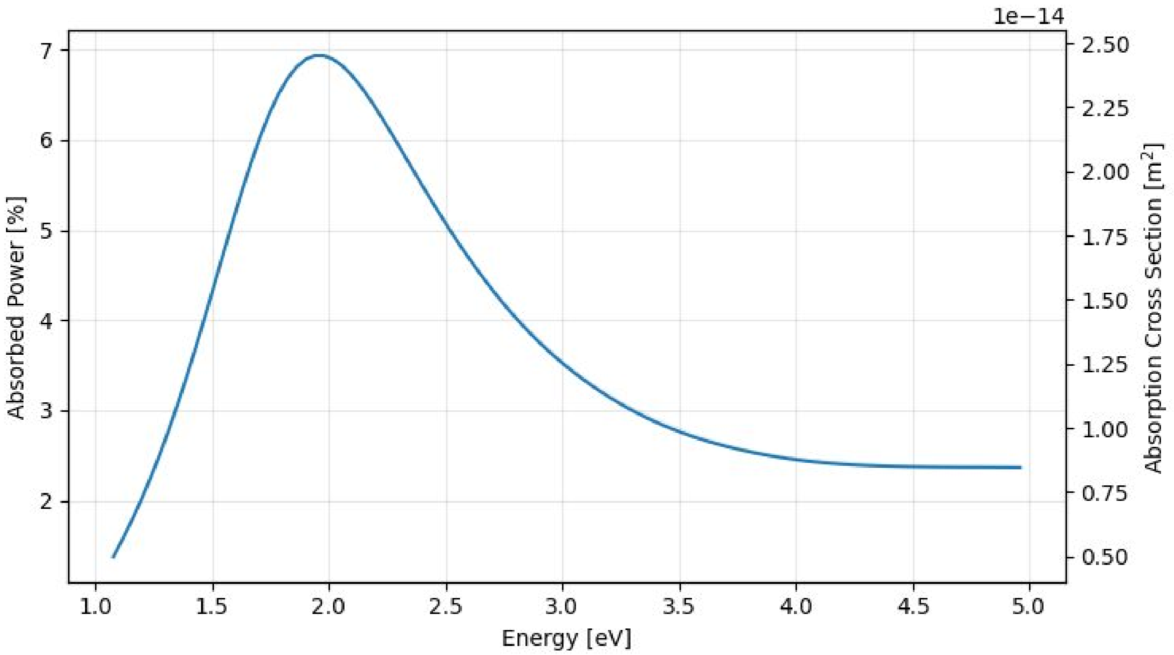

Figure S16: *The absorbed power and absorption cross section of a palladium disk with a diameter of 140 nm and a height of 25 nm on a SiO_2 support obtained from an FDTD simulation. The power is normalized to the source power and the integrated absorption is ~3.7%. The average absorption cross section is 1.32×10^{-14} m^2. The dielectric functions for Pd and SiO_2 are from Palik et al.*[4]

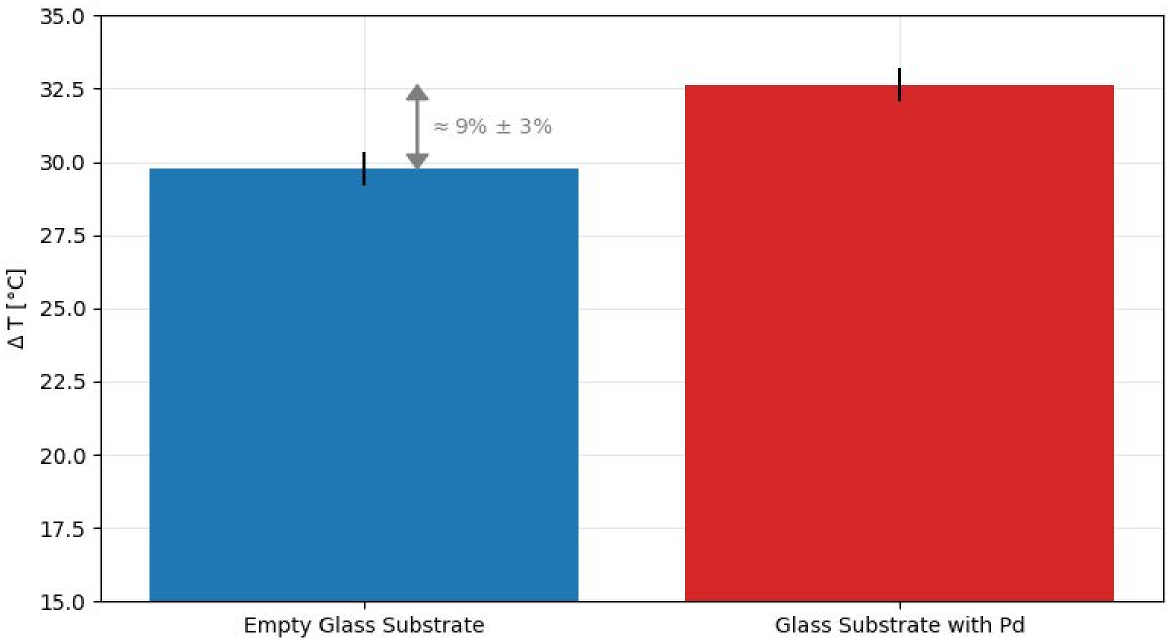

Figure S17: *Temperature increase,ΔT, measured with a thermocouple upon illumination of an empty glass substrate and a glass substrate with a Pd nanoparticle array. The measurements show that most of the heating is induced by light absorption in the reactor with only ~ 10 % stemming from the presence of the nanoparticles. Importantly, we note that this measurement only should be seen as an estimate since the exact positioning of the thermocouple has s a sizable impact of the obtained absolute numbers. The error bars are the standard deviation from 3 measurements.*

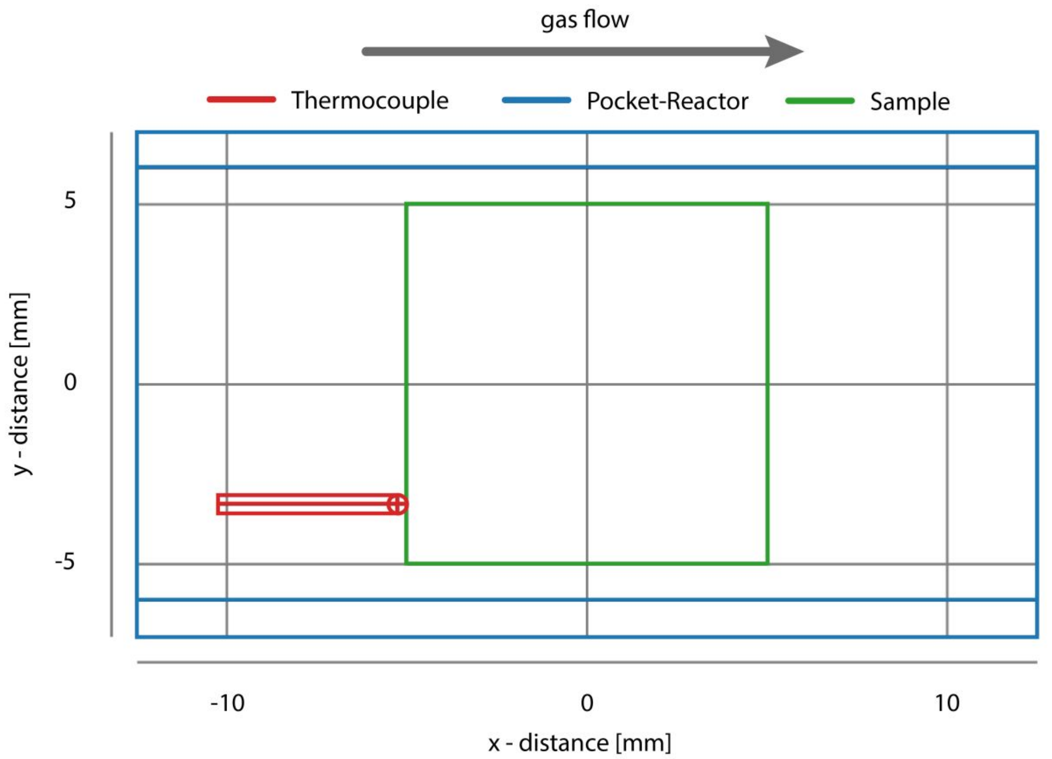

Figure S18: *The sample and setup geometry simulated in COMSOL Multiphysics projected onto 2D. The pocket reactor (blue) is 25×12×1 mm*^{3} *with a wall thickness of 1mm. The sample (green) is 9×9×0.5 mm*^{3} *and the thermocouple (red) has a diameter of 0.5 mm. The sample is placed in such a manner that it touches the pocket on its backside and has a gas flow over it from the right to the left. The sample and the pocket consist of glass, the thermocouple consists of Inconel 600 and air is used as the bypassing gas in the simulation. The material constants were taken from COMSOLs database.*

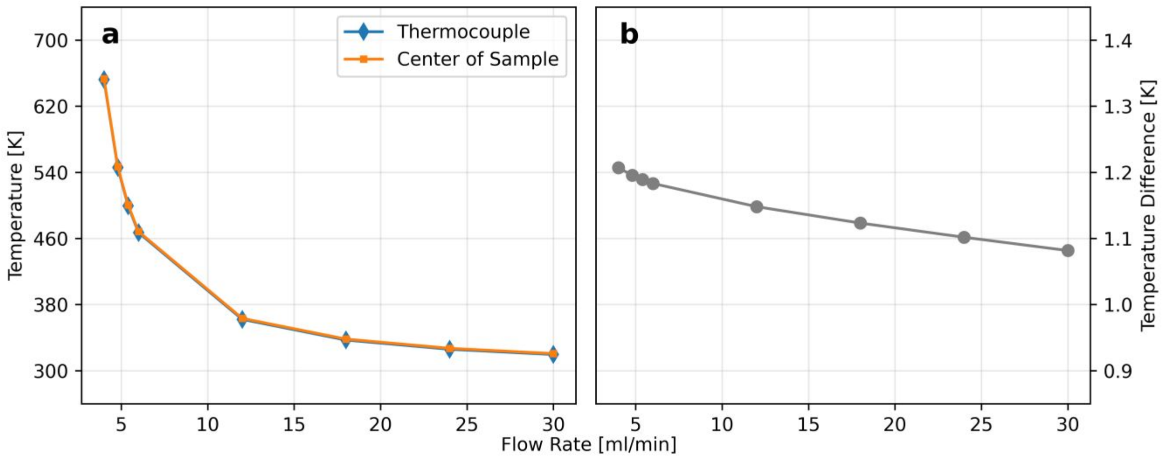

Figure S19: *a) Calculated temperature at the center and the edge of sample in air with various flow rates at 3W optical power. b) The difference between the two temperatures as a function of flow rate, which reveals a slight decrease at higher flows. Since the difference is very small, the effect of the flow rate can thus be considered negligible.*

Section S3: Photoinduced heating

To calculate the photoinduced heating we applied the theoretical model established by Baffou *et.al.*[2] using the equation for a finite square array under uniform illumination

$$\Delta T = \frac{\sigma_{\mathrm{abs}} I}{(\kappa_s + \kappa_m)/2} \frac{\ln 1 + \sqrt{2}}{\pi} \frac{S}{p^2}, \qquad \text{(S3)}$$

where ΔT is the temperature increase upon illumination, σ_{abs} is the nanoparticle absorption cross section, *I* the irradiance, κ_s and $\kappa_m$the thermal conductivity of the substrate (SiO_2) and the medium (Ar), respectively, *S* the side length of the array, and *p* the interparticle distance.

The thermal conductivity for the medium was chosen to be the one for pure Ar as our work is mostly carried out in Ar carrier gas in which H_2 is diluted, i.e., we operate in the range between 0 – 25 % H_2 only. In other words, even at the highest H_2 concentrations, it is still Ar that predominantly dictates thermal conductivity.[5]

The nanoparticle absorption cross section was obtained by FDTD calculations of a Pd disk with a diameter of 140 nm and a height of 25 nm on a SiO_2 substrate (**Figure S16**). The used source was a total-field/scattered field source with a linearly polarized plane wave and the dielectric functions for Pd and SiO_2 were obtained from Ansys-Lumericals database originating from Palik *et al.*[4]

From the estimation detailed in SI section S2 **Heat Distribution Simulations** above, we know that only ca. 10% of the heating upon illumination is caused by the particles. The rest of the heating is contributed by the rest of the setup (**Figure S17**). Therefore, here we only calculated the heating induced by the particles and we assumed that only 10% of the irradiated power reaches the sample. We used this value since it is reasonable and describes our system well.

In summary the following parameters were used:

**parameter**	**value**	**reference**
σ_{abs}	1.32×10^{-14} m^2	FDTD (**Figure S16**)
κ_s	1 W m^{-1} K^{-1}	[1]
κ_m	0.01772 W m^{-1} K^{-1}	[1]
S	9 mm	**Figure S18**
p_s	331 nm	RDF (**Figure S10**)
p_d	250 nm	RDF (**Figure S10**)

References

(1) Rumble, J. R.; Doa, M. J. *CRC Handbook of Chemistry and Physics: A Ready-Reference Book of Chemical and Physical Data*, 101st ed.; CRC Handbook of Chemistry and Physics; CRC Press: Boca Raton, Florida, 2020.

(2) Baffou, G.; Berto, P.; Bermúdez Ureña, E.; Quidant, R.; Monneret, S.; Polleux, J.; Rigneault, H. Photoinduced Heating of Nanoparticle Arrays. *ACS Nano* **2013**, *7* (8), 6478–6488. https://doi.org/10.1021/nn401924n.

(3) Palm, K. J.; Murray, J. B.; Narayan, T. C.; Munday, J. N. Dynamic Optical Properties of Metal Hydrides. *ACS Photonics* **2018**, *5* (11), 4677–4686. https://doi.org/10.1021/acsphotonics.8b01243.

(4) Palik, E. D. *Handbook of Optical Constants of Solids*, Vol. 1-3.; Academic press: Orlando, 1998.

(5) Zhukov, V. P.; Pätz, M. On Thermal Conductivity of Gas Mixtures Containing Hydrogen. *Heat Mass Transf. und Stoffuebertragung* **2017**, *53* (6), 2219–2222. https://doi.org/10.1007/s00231-016-1952-9.
